# Spatial-Jitter Model for Magnetoencephalography Sensor Arrays

**DOI:** 10.1109/TMI.2026.3687982

**Published:** 2026-07

**Authors:** Joonas Iivanainen

**Affiliations:** Department of Neuroscience and Biomedical Engineering, Aalto University, 02150 Espoo, Finland

**Keywords:** Magnetoencephalography, optically pumped magnetometer, SQUID, position error, jitter, magnetic field

## Abstract

Sampling jitter, i.e., random deviations in the time instants when samples are taken, causes frequency-dependent noise that reduces signal-to-noise ratio (SNR). This paper generalizes the concept of jitter to magnetoencephalography (MEG) sensor arrays that spatially sample the quasistatic magnetic field due to brain activity. It is shown that spatial jitter, i.e., random deviations in MEG sensor positions, causes spatial-frequency-dependent noise in the vector spherical harmonics domain that reduces the attainable SNR and spatial resolution in MEG. Similarly, the paper also considers noise due to random sensor orientation errors (‘orientation jitter’) and errors due to field integration by the finite-sized sensors (‘aperture error’). The analysis in this paper shows that on-scalp MEG measurements taken closer to the head are more resistant to spatial and orientation jitter at high spatial frequencies than off-scalp measurements taken further away. On the other hand, on-scalp measurements are affected more by aperture errors than off-scalp measurements. The paper also provides new insights to the effect of sensor noise on the spatial resolution of on- and off-scalp sensor arrays using a novel normalization of the vector spherical harmonics. The paper also simulates spatial-jitter phenomena with realistic sensor arrays based on optically pumped magnetometers and superconducting quantum interference device sensors. This realistic simulation shows that spatial jitter reduces SNR and affects how the measurements should be regularized in order to maximize SNR.

## I. Introduction

Magnetoencephalography (MEG) is a noninvasive neuroimaging technique in which the weak magnetic fields of the human brain are detected outside the head [1], [2]. Until recently, MEG systems have relied on superconducting quantum interference device (SQUID) sensors to measure the neuromagnetic field, but MEG is undergoing transformation facilitated by the development of a new sensor technology. The optically pumped magnetometer (OPM) allows one to measure the neuromagnetic fields closer to the head than the SQUID sensors (about 4 mm vs. > 2 cm) albeit with a worse sensitivity (10)–20 $\text{fT}∕\sqrt{\text{Hz}}$ vs. 2–3 $\text{fT}∕\sqrt{\text{Hz}}$, respectively) [3].

The benefits of OPM-based ‘on-scalp’ MEG measurements over SQUID-based ‘off-scalp’ measurements have been largely established by numerous simulation studies. OPM-based arrays offer improvements over SQUID-arrays in terms of signal amplitude, spatial resolution of the measurements, as well as their information content [4], [5], [6], [7]. The ability to measure two or all three orthogonal components of the field with OPM sensors compared to sampling a single field component with single-axis SQUID sensors provides improvements in interference rejection [8], [9]. In addition, studies have estimated the required number of OPM sensors to adequately spatially sample the field and methods to optimally construct OPM-based sensor arrays have been introduced [6], [7], [10], [11], [12], [13], [14], [15], [16]. While these studies provide the backbone of the expected performance improvements of on-scalp arrays over off-scalp arrays, the mechanisms that could limit their actual real-world performance have not been considered as widely.

For high-resolution source imaging, the sensor positions and orientations need to be accurately known with respect to each other and the subject’s anatomy. To this end, many studies have directly quantified the impact of sensor position errors on MEG source imaging in on-scalp sensor arrays [17], [18], [19], [20]. However, an overarching theoretical model underlying the effects of these errors on MEG measurements is still lacking.

This paper sets out to develop an approach based on first principles to assess the effect of random sensor position errors on the attainable signal-to-noise ratio (SNR) and spatial resolution in MEG. Random sensor position errors (‘spatial jitter’) are defined as random errors in the positions of the individual sensors that are not correlated across the sensors. The central argument of the paper is that random sensor position errors affect the spatial sampling of the quasistatic neuromagnetic field similarly as jitter affects temporal signal acquisition: they both cause frequency-dependent noise that degrades SNR and limits the measurement resolution. In addition to spatial jitter, the paper considers other error sources such as noise due to random sensor orientation errors (‘orientation jitter’) and errors due to field integration by the sensor (‘aperture errors’). The sensitivity of both on-scalp and off-scalp MEG measurements to these error sources is analyzed.

## II. Theoretical Background

Here, the theoretical background of the spatial-jitter model is introduced. It is first presented how jitter and finite aperture time affect the sampling of temporal signals in one dimension. These concepts are then related to spatial sampling of the quasistatic magnetic field.

### A. Jitter and Aperture Errors in One-Dimensional Sampling

The following introduction on jitter and finite aperture errors in one-dimensional sampling is based on the paper by Kobayashi and colleagues [21]. Suppose the following sinusoidal input:

(1) $$V_{\text{in}}(t)=A\;\cos\;(2\pi f_{\text{in}}t),$$

where A and $f_{\text{in}}$ are the amplitude and frequency of the sinusoid, respectively. Let us assume that the system samples the signal at time instants $t=nT_{s}+\epsilon_{n}$, where $T_{s}$ is the average sampling period and $\epsilon_{n}$ is the sampling jitter that follows a Gaussian distribution $N(0,\sigma^{2})$. The output of the sampling system is then $V_{\text{out}}(nT_{s})=V_{\text{in}}(nT_{s}+\epsilon_{n})$. Kobayashi and colleagues showed that the noise power due to jitter in this case is [21]

(2) $$P_{N}=A^{2}[1-\text{exp}(-2\pi^{2}f_{\text{in}}^{2}\sigma^{2})].$$

Since the signal power of the sinusoid is $P_{S}=A^{2}∕2$, the SNR after sampling is

(3) $$\text{SNR}=\frac{P_{S}}{P_{N}}=\frac{1}{2(1-\text{exp}(-2\pi^{2}f_{\text{in}}^{2}\sigma^{2}))}.$$

Noise due to jitter is frequency dependent: as the frequency of the input sinusoid increases, the SNR progressively decreases at a constant level of jitter σ.

Next, let us go through the corresponding result for a general input signal that can be represented through its Fourier transform

(4) $$V_{\text{in}}(t)=\frac{1}{2\pi }\int_{-\infty }^{\infty }F(j\omega )\;{\text{exp}}^{-j\omega t}d\omega ,$$

where ω is the angular frequency. According to Parseval’s theorem, the input signal power is equal in time and spectral (frequency) domains [22]:

(5) $$P_{S}=\int_{-\infty }^{\infty }{|V_{\text{in}}(t)|}^{2}dt=\frac{1}{2\pi }\int_{-\infty }^{\infty }{|F(j\omega )|}^{2}d\omega .$$

For a general input signal, the noise power due to jitter is [21]

(6) $$P_{N}=\frac{1}{\pi }\int_{-\infty }^{\infty }{|F(j\omega )|}^{2}\left(1-\text{exp}\left(-\frac{\omega^{2}\sigma^{2}}{2}\right)\right)d\omega ,$$

and the SNR is the ratio of input and noise power in the frequency domain. The jitter noise power depends on both the frequency and the signal energy at that frequency.

In addition to jitter, there is another source of error in the sampling system. Ideally, sampling should be performed instantaneously at a time t. However, typically the sampling instead takes a finite amount of time Δt and the output of the system is given by the convolution [21]

(7) $$V_{\text{out}}(t)=\int_{-\Delta t∕2}^{\Delta t∕2}g(\tau )V_{\text{in}}(t+\tau )d\tau ,$$

where g(τ) is the aperture function representing how the system integrates its input. In the case that both jitter and finite aperture are taken into account, the total noise power is [21]

(8) $$P_{N}=\frac{1}{2\pi }\int_{-\infty }^{\infty }{|F(j\omega )|}^{2}[{|1-G(j\omega )|}^{2}\;\;+2G_{R}(\omega )\left(1-\text{exp}\left(-\frac{\omega^{2}\sigma^{2}}{2}\right)\right)]d\omega ,$$

where G(jω) is the Fourier transform of the aperture function g(τ) and $G_{R}(\omega )$ is its real part. Eq. (8) shows that the total noise power can be written as: (error power due to finite aperture) $+G_{R}(\omega )\times$ (noise power due to jitter). Thus, the jitter noise gets multiplied by the aperture transfer function. In other words, the sensor aperture, in addition to causing its own error, affects how jitter noise maps to the overall noise.

### B. Quasistatic Magnetic Field and Its Representation With Vector Spherical Harmonics

This section considers a case where we have a current distribution inside a volume and we use an instrument to measure its quasistatic magnetic field outside the volume. The quasistatic magnetic field in the source-free volume can be represented as [23], [24]

(9) $$\vec{B}(\vec{r})=-\mu_{0}\sum_{l=1}^{\infty }\sum_{m=-l}^{l}\alpha_{lm}\sqrt{\left(l+1)(2l+1\right)}\frac{{\vec{V}}_{lm}(\theta ,\phi )}{r^{l+2}},$$

where (r, θ, ϕ) are the spherical coordinates, $\mu_{0}$ is the permeability of free space, ${\vec{V}}_{lm}$ are the vector spherical harmonics (VSHs) and $\alpha_{lm}$ are their coefficients. The VSHs are orthonormal with respect to the inner product of integrating over the solid angle

(10) $$\int_{\Omega }{\vec{V}}_{lm}(\theta ,\phi )\cdot {\vec{V}}_{l^{\prime }m^{\prime }}^{*}(\theta ,\phi )d\Omega =\delta_{ll^{\prime }}\delta_{mm^{\prime }},$$

where ⋅ is the dot product and $\delta_{ll^{\prime }}$ is the Kronecker delta. The magnetic field representation in Eq. (9) converges at all points that are outside a sphere that encapsulates all the sources [25]. The representation thereby also applies to non-spherical geometries as long as the previous holds true.

The normalization of the VSH coefficients and the basis functions in Eq. (9) is arbitrary. Here, it is proposed to normalize the VSH functions to one unit of magnetic energy (see Appendix A). This means that the magnetic field in Eq. (9) is written as

(11) $$\vec{B}(\vec{r})=\sum_{l=1}^{\infty }\sum_{m=-l}^{l}a_{lm}{\vec{b}}_{lm}(\vec{r}),$$

for which the magnetic energy can be written as (Appendix A)

(12) $$E=\frac{1}{2}\int_{r>R}\frac{B^{2}}{\mu_{0}}dV=\frac{1}{2}\sum_{l,m}{|a_{lm}|}^{2},$$

where R is the radius of a sphere that encapsulates all the sources, and the vectors

(13) $${\vec{b}}_{lm}(\vec{r})=-\sqrt{\mu_{0}R^{2l+1}(2l+1)}\frac{{\vec{V}}_{lm}(\theta ,\phi )}{r^{l+2}},$$

are orthonormal with respect to the energy integral. The units are so that ${|a_{lm}|}^{2}$ are in joules and $a_{lm}{\vec{b}}_{lm}(\vec{r})$ are in teslas. Eq. (12) is essentially the Parseval’s theorem (see Eq. (5)) for magnetic field stating that the magnetic energy is the same in the spatial and spectral (VSH) domains. Let us further define the energy-spectral density (ESD) of the magnetic field as a function of l as

(14) $$\text{ESD}(l)=\sum_{m=-l}^{l}{|a_{lm}|}^{2}.$$

### C. The Analogy Between Sampling of One-Dimensional Signals and Quasistatic Magnetic Fields

Although spatial sampling of magnetic field is different from sampling temporal signals in one dimension, this paper argues that they are conceptually similar and subject to the same noise sources in a similar fashion. In the following sections, this is shown with simulations. Here, an intuitive analogy between one-dimensional signal and magnetic field sampling is built.

The magnetic field representation in Eq. (11) is analogous to the Fourier representation of a one-dimensional signal in Eq. (4): it is a hierarchical representation of the signal in terms of frequency. The analogy between these two representations follows from the fact that their basis functions are eigenfunctions of the negative Laplace operator on their respective domains:

(15) $$-\nabla^{2}f=k^{2}\;f,$$

where f is the basis function and k is the wavenumber related to (spatial) frequency. For Fourier basis, the domain is the infinite line with f=exp(−jωt) and k=ω. For the magnetic field representation, the domain is a sphere, and $f=Y_{lm}$ are the spherical harmonics and $k=\sqrt{l(l+1)}$. The magnetic field scalar potential is cast as a function of $Y_{lm}$ and the vector presentation in Eq. (11) is obtained by taking the gradient of the scalar potential.

While the one-dimensional signal energy can be decomposed into a continuum of frequencies (see the integral in the Fourier representation in Eq. (4)), the magnetic field energy can be decomposed into discrete spatial frequencies characterized by the degrees l of the spherical harmonics (see the sum in Eq. (11)). Each one-dimensional frequency corresponds to two phase components (sine and cosine), which both are eigenfunctions of $-\nabla^{2}$. Each spherical harmonics degree l related to a spatial frequency corresponds to 2l+1 phase components characterized by the order m of the spherical harmonics.

Using this analogy, it is expected that spatial jitter, i.e., random sensor position errors in the measurement array, leads to noise that is dependent on the spatial frequency and the signal energy at that frequency as in Eq. (6). This concept can be generalized to also define orientation jitter, i.e., random errors in the sensing axes of our measurement sensors.

Similarly, aperture effects from temporal sampling can be generalized to spatial magnetic field sampling. The output of a magnetic sensor at $\vec{r}$ is given by the integral [26], [27], [28]

(16) $$b(\vec{r})=\int \vec{g}({\vec{r}}^{\prime })\cdot \vec{B}(\vec{r}+{\vec{r}}^{\prime })dV^{\prime }\;\;=\sum_{l=1}^{\infty }\sum_{m=-l}^{l}a_{lm}\int \vec{g}({\vec{r}}^{\prime })\cdot {\vec{b}}_{lm}(\vec{r}+{\vec{r}}^{\prime })dV^{\prime },$$

where $\vec{g}(\vec{r})$ is aperture function of the sensor representing how the sensor integrates the magnetic field. Typically, the sensor aperture function is a boxcar function as the sensor uniformly integrates the magnetic field in its volume. Wider uniform sensor apertures integrate the magnetic field in a larger area and lead to a more pronounced spatial low-pass filtering by the sensor due to spatial averaging. This increases aperture errors at high spatial frequencies as the signal is attenuated at these frequencies. In principle, the aperture function could also be more complicated to represent non-uniform weighting of the magnetic field or integration of different field components within a sensor. It is expected that in addition to the sensor aperture causing its own error it will affect how spatial and orientation jitter noise map to the sensor array output (Eq. (8)). Please note that here it is referred to aperture errors and to jitter noise due to the latter being stochastic.

Similar to the SNR definition in one dimension, let us define SNR as a function of the spatial band limit $l_{B}$ as the square root of the ratio of the total signal energy and the total noise:

(17) $$\text{SNR}(l_{B})=\sqrt{\frac{\sum_{l=1}^{l_{B}}\sum_{m=-l}^{l}{|a_{lm}^{S}|}^{2}}{\sum_{l=1}^{l_{B}}\sum_{m=-l}^{l}{|a_{lm}^{N}|}^{2}}},$$

where $a_{lm}^{S}$ and $a_{lm}^{N}$ are the VSH coefficients of the signal and noise, respectively. Square root is taken to convert the SNR to amplitude ratio. The spatial bandlimit is analogous to that in one dimension. The bandlimit controls the maximum spatial frequency and, thereby, also the spatial resolution of the measurements.

Let us further define the transfer function of the measurement as a function of the spherical harmonics degree as

(18) $$\text{TF}(l)=\frac{\sum_{m=-l}^{l}{|a_{lm}^{\text{out}}|}^{2}}{\sum_{m=-l}^{l}{|a_{lm}^{S}|}^{2}},$$

where $a_{lm}^{S}$ are the VSH coefficients of the signal (the source) magnetic field and $a_{lm}^{\text{out}}$ are the VSH coefficients after measurement with the array.

## III. Methods

**Fig. 1.**
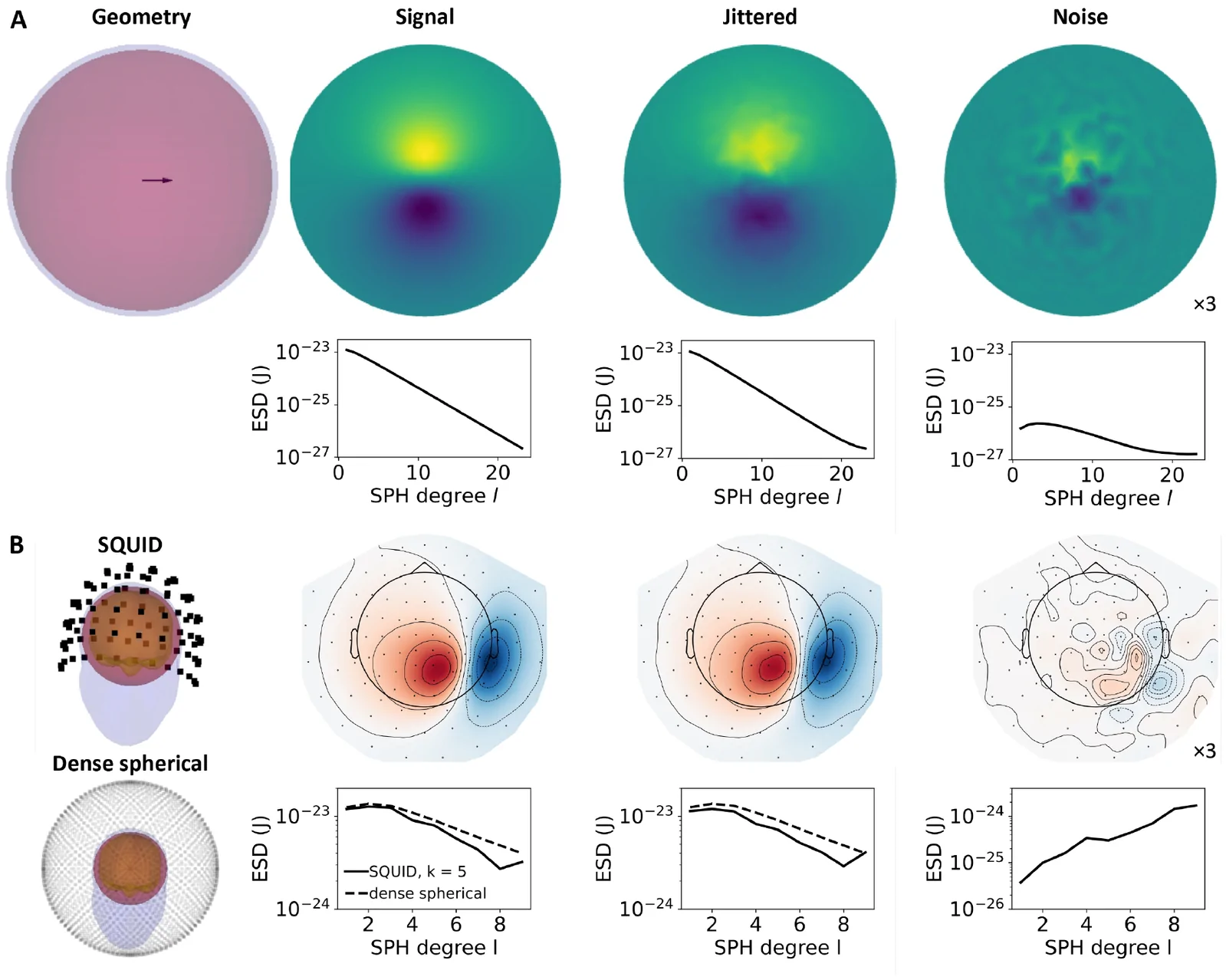
Illustration of the simulation pipeline for spherical (A) and realistic (B) geometry. **A:** The magnetic field of a tangential current dipole inside a spherical conductor is evaluated at a dense spherical sensor array (the signal). Spatial jitter (random sensor displacements) is added and the field is evaluated at the jittered sensor positions. Noise due to spatial jitter is obtained by subtracting the signal from the jittered sensor array output. The lower part of the panel shows the energy-spectral densities (ESDs) of the signal and noise as a function of the spherical harmonics (SPH) degree l. **B:** As in panel A, but for a 102-sensor SQUID magnetometer array using a current dipole in a realistic head model. The field is also evaluated at a dense spherical sensor array to obtain the ‘ground-truth’ ESD. The regularization exponent k=5 in the least-squares estimation of the spherical multipole moments is chosen so that it minimizes the estimation error between the SQUID and ground-truth ESD. The noise patterns are multiplied by a factor of three for visualization.

The spatial-jitter model is demonstrated using simulations both in spherical and realistic volume conductors using both dense spherical and realistic sensor arrays. Fig. 1 illustrates the simulation pipelines for the spherical and realistic geometries.

### A. Spherical Conductor

The magnetic fields of tangential current dipoles inside a spherical volume conductor are simulated using the Sarvas formula [29]. The radius of the sphere is set to 9.2 cm [30], [31]. The moments of the current dipoles are set to 10 nAm. Unless stated otherwise, the simulations are conducted for a 1.7-cm deep dipole.

The magnetic field is calculated at 2,702 measurement points on a sphere. Each point has a virtual sensor with three orthogonal measurement directions. For ‘on-scalp’ measurements, the measurement points are at a distance of 4 mm from the surface of the spherical conductor. For ‘off-scalp’ measurements, the points are at a distance of 2 cm. The on- and off-scalp measurements are not to be confused with realistic sensor arrays, but the distances are set to according to those obtained with OPMs and SQUIDs. A large number of measurement points is used to get accurate estimates on how spatial jitter affects the VSH coefficients at these distances from the head. The sensor arrays cover the whole spherical surface and the sensor positions are defined according to the Lebedev quadrature for integrating a function over the surface of a sphere [32]. Using the Lebedev quadrature, VSH inner product of Eq. (10) is discretized as

(19) $$\int_{\Omega }{\vec{V}}_{lm}(\theta ,\phi )\cdot {\vec{V}}_{l^{\prime }m^{\prime }}^{*}(\theta ,\phi )d\Omega \approx \sum_{i=1}^{N_{q}}w_{i}{\vec{V}}_{lm}({\vec{r}}_{i})\cdot {\vec{V}}_{l^{\prime }m^{\prime }}^{*}({\vec{r}}_{i}),$$

where ${\vec{r}}_{i}$ and $w_{i}$ are the quadrature points and weights, respectively, and $N_{q}$ is their number.

The VSHs are generated using the bfieldtools software package with normalization option set to “energy” [25], [33]. The VSH coefficients are estimated using the discretized inner product as:

(20) $$a_{lm}=-\frac{R_{m}^{2l+4}}{\mu_{0}(2l+1)R^{2l+1}}\sum_{i=1}^{N_{q}}w_{i}\vec{y}({\vec{r}}_{i})\cdot {\vec{b}}_{lm}({\vec{r}}_{i}),$$

where $R_{m}$ is the radius of the measurement sphere, R is the radius of the spherical conductor and $\vec{y}({\vec{r}}_{i})$ is the simulated magnetic field at ${\vec{r}}_{i}$. The expansion order of the VSHs is set to l=23. ESD is calculated as a function of spherical harmonics degree l by squaring the multipole moments and summing over m for each l (Eq. (14)).

Different error sources are simulated as follows. Spatial white noise (representing sensor noise) is simulated by adding to the measurements normally distributed random values that are independent across measurement points and axes. The standard deviation of the normal distribution is $\sigma_{s}\sqrt{\text{BW}}$, where $\sigma_{s}$ is the sensor white noise level in $\text{fT}∕\sqrt{\text{Hz}}$ and BW is the measurement bandwidth that is assumed to be 40 Hz.

Spatial jitter is simulated by adding random displacements to the measurement positions. The random displacement at each measurement position is drawn uniformly from a half sphere oriented along the positive radial direction. Random displacements along the negative radial direction are not included as the measurement positions could end up inside the spherical conductor, which is physically unfeasible. The effect of spatial jitter along the radial direction and on the tangential plane are also simulated separately. For tangential spatial jitter, the random displacements are drawn from a circle on the tangential plane. Spatial jitter of d mm is defined as the radius of the half sphere or the circle. Orientation jitter is likewise simulated by adding random perturbations following a zero-mean uniform distribution to the measurement axes. Sensor aperture is modeled by discretizing Eq. (16): the field is averaged over four integration points at the tangent plane defined around the measurement point by (±Δ∕4,±Δ∕4), where Δ is the sensor dimension. In addition to sensor-wise spatial jitter, errors due to translations and rotations of the whole sensor array are simulated by applying random rotation matrices and translation vectors that act on all the measurement points simultaneously.

The noise multipole moments are estimated by $a_{lm}^{N}=a_{lm}^{\text{meas}}-a_{lm}^{S}$, where $a_{lm}^{\text{meas}}$ are the multipole moments of the measurement including the source magnetic field and sensor and jitter noise and $a_{lm}^{S}$ are the multipole moments of the source magnetic field without any errors. The noise ESD is computed as an average over 50 simulations of random draws.

### B. Realistic Geometry

Spatial-jitter simulations are also conducted in a realistic geometry using the anatomical data from the MNE sample subject from the MNE Python software, and using methods for magnetic-field calculation implemented in that software [34]. MRI data for this subject were acquired with a Siemens 1.5-T Sonata scanner using an MPRAGE sequence. The primary current distribution on the two hemispheres is discretized to 7,498 current dipoles oriented normal to the local cortical surface. A single-layer boundary element method model of the head (conductivity: 0.3 S/m; number of vertices: 2,562) is used to calculate the magnetic field of the current dipoles.

The field is calculated for four sensor arrays: 102-sensor SQUID magnetometer array (MEGIN Oy, Espoo, Finland), 102-sensor single-axis OPM array measuring the field component normal to the subject’s head surface, 102-sensor triaxial OPM array (TriOPM; 306 channels) and a spherical sensor array with 2,702 triaxial point-like magnetometers. The geometry of the OPMs is modeled according to the Gen-1 OPM by QuSpin Inc. (Louisville, CO, USA). The magnetic field integration due to the sensor aperture is modeled as in the MNE-Python software. The positions of the SQUID sensors with respect to the subject’s anatomy are taken as in the dataset representing the sensor positions in an MEG experiment. OPM sensor positions are obtained by projecting the SQUID sensors towards the head so that their average distance to the head is about 6 mm. The radius of the spherical sensor array is set to about 18.6 cm so that it is completely outside the head. Its origin is set at the average coordinates of the brain surface translated 1 cm towards the neck. This same origin is also used in the VSH expansion. The radius of the VSH expansion sphere is set to about 8.2 cm. The VSH expansion order is set to l=9 so that the number of components is as large as possible, but less than the smallest number of channels in the sensor arrays (99 components vs. 102 channels).

For the dense spherical sensor array, the discretized inner product of Eq. (20) is used to calculate the VSH coefficients. These coefficients represent the ‘ground truth’ as they are obtained at dense spherical measurement points. For the OPM and SQUID arrays, the VSH coefficients are estimated using the regularized least-squares method [33]:

(21) $$a={\left(S^{T}S+\lambda L\right)}^{-1}S^{T}y,$$

where a is the vector of the estimated l(l+2) VSH multipole moments, y are the measurements by N channels, S is a N×l(l+2) matrix relating the measurements to the multipole moments with the basis functions ${\vec{b}}_{lm}(\vec{r})$ (Eq. (13)) as columns, L is the surface Laplacian on the sphere and λ is the regularization parameter.

Spatial jitter was simulated by adding random displacements to the sensor positions similarly as in the simulations in the spherical conductor. The noise multipole moments were computed by subtracting the estimated moments from those obtained with the dense spherical array. The noise ESD and SNR at bandlimit $l_{B}=9$ were calculated as an average over 50 iterations. To study the effect of regularization on the obtained SNR, the regularization parameter was varied as $\lambda =10^{-k}\times \sigma_{\max}$, where k is between 1–9 and $\sigma_{\max}$ is the maximum eigenvalue of ${\mathrm{SS}}^{T}$.

Simulations were also performed for the 102-sensor SQUID array by including two noise models in addition to spatial jitter. In the first case, the noise was modeled as sensor white noise with a level of 3 $\text{fT}∕\sqrt{\text{Hz}}$. In the second case, noise covariance matrix estimated from data was incorporated. For this purpose, data were measured with 102 SQUID magnetometers of a commercial MEG scanner (MEGIN Oy; the same scanner as in other SQUID simulations) inside an empty three-layer magnetically shielded room (Imedco AG, Hägendorf, Switzerland). Spatial-jitter simulations were conducted in a similar fashion as described previously, but sensor and empty-room noise were included. The bandwidth was set to 40 Hz and the simulations were performed using source amplitudes of 10 and 1 $\mu {\text{Am}}_{\text{rms}}$.

## IV. Results

Results are generally shown as a function of l, but for some simulations a bandlimit needs to be chosen; for this purpose, the results are generally shown at bandlimits $l_{B}=8$ and $l_{B}=16$.

### A. Spatial White (sensor) Noise

**Fig. 2.**
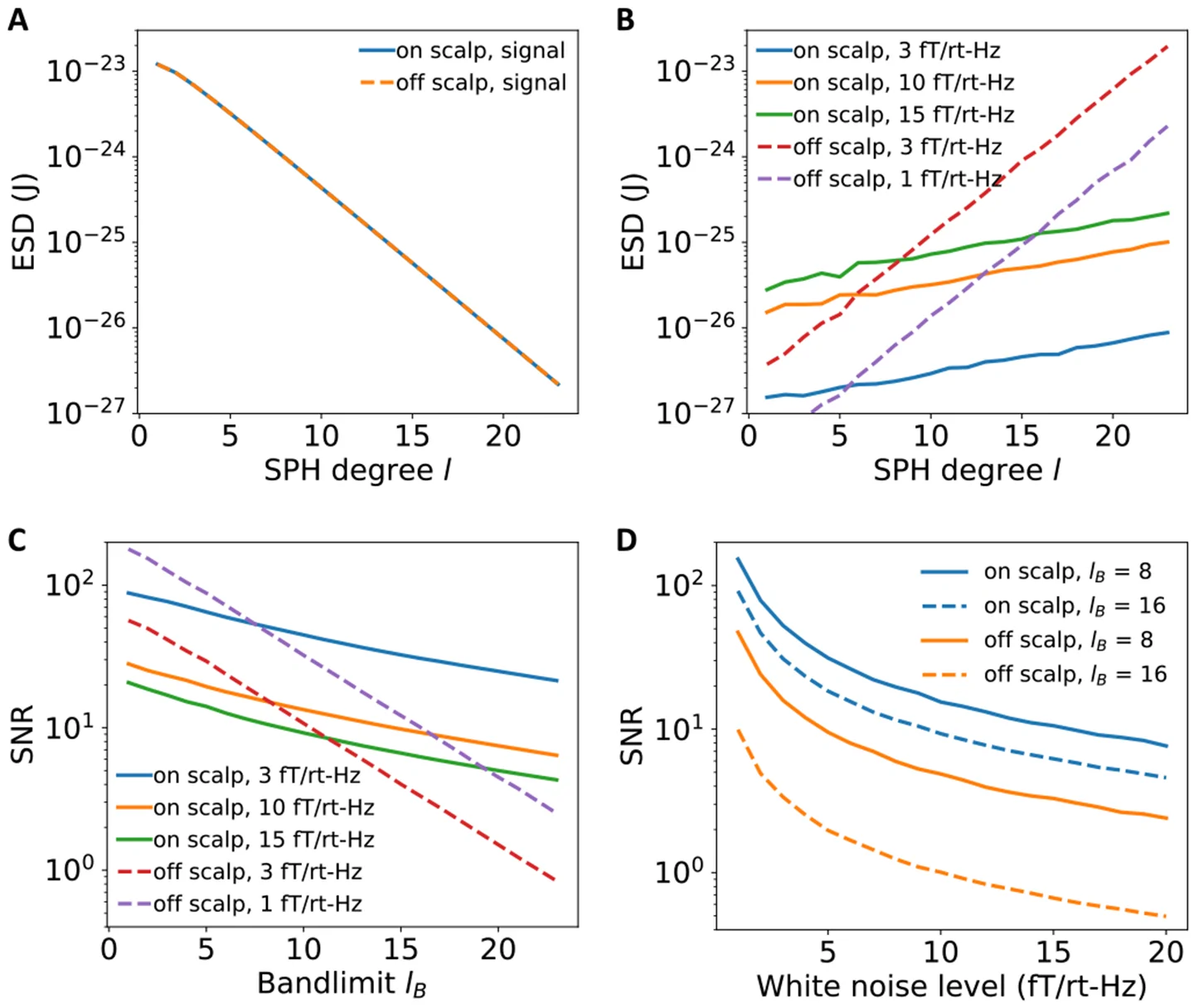
Energy-spectral density (ESD) of signal and spatial white noise in the vector-spherical harmonics domain. The ESD is in joules (J). **A:** ESDs of the magnetic field of a tangential current dipole inside a spherical conductor (the signal) at two different distances from the sphere surface (on scalp: 4 mm; off scalp: 20 mm). **B:** ESDs of different levels of spatial white noise (independent across measurement points and axes) for on- and off-scalp measurements. **C:** Signal-to-noise ratio (SNR) of the signal and noise presented in panels A and B, respectively, as a function of the bandlimit for the spherical harmonics degree $l_{B}$. **D:** SNR as a function of the spatial white noise level at bandlimits of $l_{B}=8$ and $l_{B}=16$.

First, the effect of spatial white noise on the VSH spectral density and the measurement SNR (Eq. (17)) is simulated. This kind of noise is uncorrelated across the measurement points and directions; it can be thus taken to represent, e.g., sensor noise. Fig. 2A shows the ESDs of the signal (the magnetic field from the source current dipole) for on- and off-scalp arrays. The ESDs show an important aspect about the VSH normalization applied in this work: both on- and off-scalp sensor arrays recover the same ESD (and the same VSH coefficients).

Fig. 2B shows the noise ESDs due to different levels of spatial white noise (or sensor noise). This kind of noise leads to l-dependent noise on the ESD that reduces the SNR of the measurement of the magnetic field of the source dipole. Using the normalization scheme proposed here, the same amount of spatial white noise in on- and off-scalp arrays leads to different noise ESDs; the noise ESD increases faster as a function of l in off-scalp than in on-scalp measurements.

Fig. 2C shows the SNR as a function of the spherical harmonics bandlimit $l_{B}$ (see Eq. (17)). With the same sensor noise level of 3 $\text{fT}∕\sqrt{\text{Hz}}$, the on-scalp measurement SNR is higher than that of off-scalp measurement. With higher sensor noise in on-scalp measurements (≥ 10 $\text{fT}∕\sqrt{\text{Hz}}$), the off-scalp SNR is initially higher than the on-scalp SNR, but the off-scalp SNR reduces with a steeper slope than that in on-scalp measurements as the spatial bandlimit increases. At on-scalp noise levels of 10 and 15 $\text{fT}∕\sqrt{\text{Hz}}$, the crossings of on- and off-scalp SNR occur at around $l_{B}=8$ and $l_{B}=11$, respectively. Fig. 2D shows how the SNR behaves as a function of sensor noise at $l_{B}=8$ and $l_{B}=16$.

**Fig. 3.**
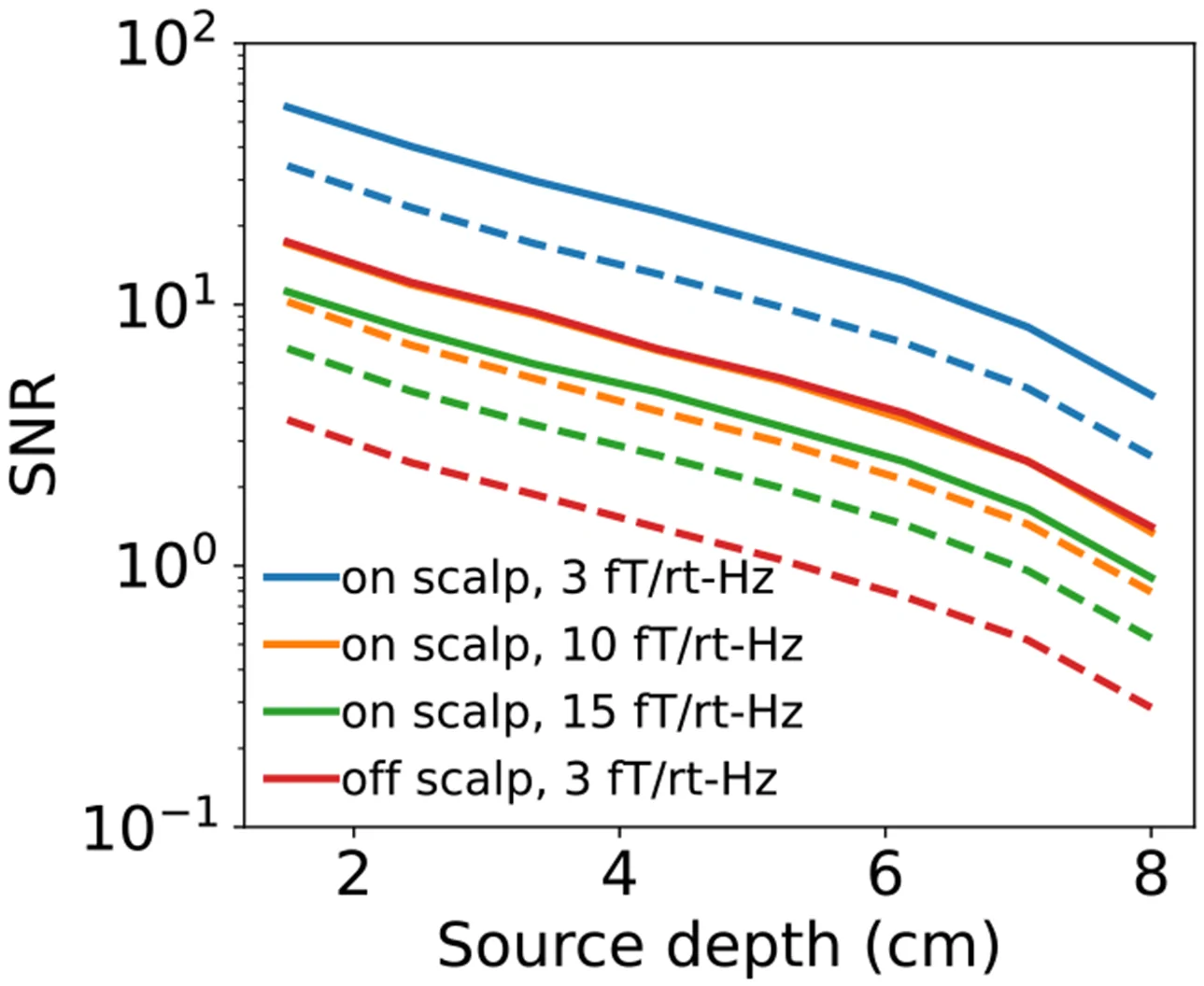
Signal-to-noise ratio (SNR) of on- and off-scalp magnetic measurements of a tangential current dipole as a function of dipole depth in a spherical conductor. Solid lines: spherical harmonics bandlimit $l_{B}=8$; dashed lines: $l_{B}=16$.

Fig. 3 shows the SNR as a function of source depth at various noise levels at bandlimits $l_{B}=8$ and $l_{B}=16$. The SNR decreases as source depth increases. At $l_{B}=8$, on-scalp measurements with a noise level of 10 $\text{fT}∕\sqrt{\text{Hz}}$ give an equivalent SNR to off-scalp measurement at 3 $\text{fT}∕\sqrt{\text{Hz}}$. The off-scalp SNR reduces more vs. on-scalp SNR as a function of the bandlimit: at $l_{B}=16$, on-scalp SNR with a noise level of 10 $\text{fT}∕\sqrt{\text{Hz}}$ is about three times better than that of off-scalp measurements. Even with a noise level of 15 $\text{fT}∕\sqrt{\text{Hz}}$, the on-scalp SNR is two times better than that of off-scalp measurements at $l_{B}=16$.

### B. Spatial and Orientation Jitter

**Fig. 4.**
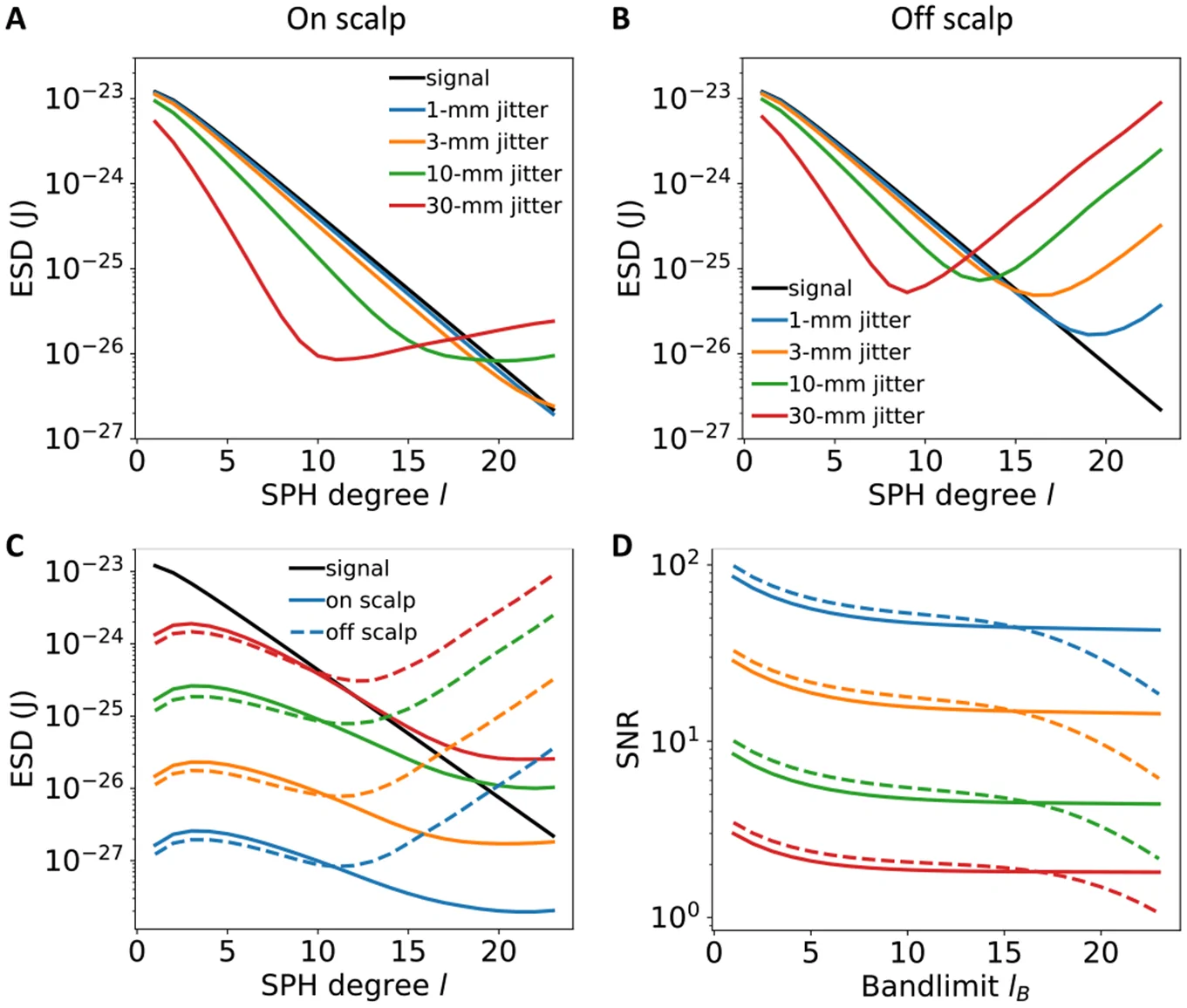
Noise caused by spatial jitter, i.e., random sensor position errors. **A:** ESD of the on-scalp measurement of a magnetic field of a tangential current dipole at different levels of spatial jitter. **B:** As in A, but for off-scalp measurement. **C:** ESDs of spatial-jitter noise in relation to the signal ESD. Solid lines: on scalp; dashed lines: off scalp. The curves are colored as in panels A and B. **D:** SNRs of on-and off-scalp measurements as a function of the spherical harmonics bandlimit at different levels of spatial jitter.

Figs. 4A and B show the ESDs of on- and off-scalp measurements when different levels of spatial jitter are added to the measurement positions. As the spatial jitter increases, the ESD levels change with respect to the errorless signal ESD, this effect being more pronounced in the off-scalp measurements. The change in the ESD is due to the noise (error) caused by the spatial jitter. Fig. 4C shows the ESDs of the spatial-jitter noise. Spatial jitter causes spatial-frequency (l) dependent noise. This type of noise is slightly lower for off-scalp measurements than for on-scalp measurements at low spatial frequencies; however, the noise increases faster for off-scalp array at high l-values. Fig. 4D shows the measurement SNRs as a function of the bandlimit $l_{B}$ and spatial-jitter level. The off-scalp SNR is initially slightly higher than that of on-scalp arrays at bandlimits $l_{B}<16$ but decreases faster after that.

**Fig. 5.**
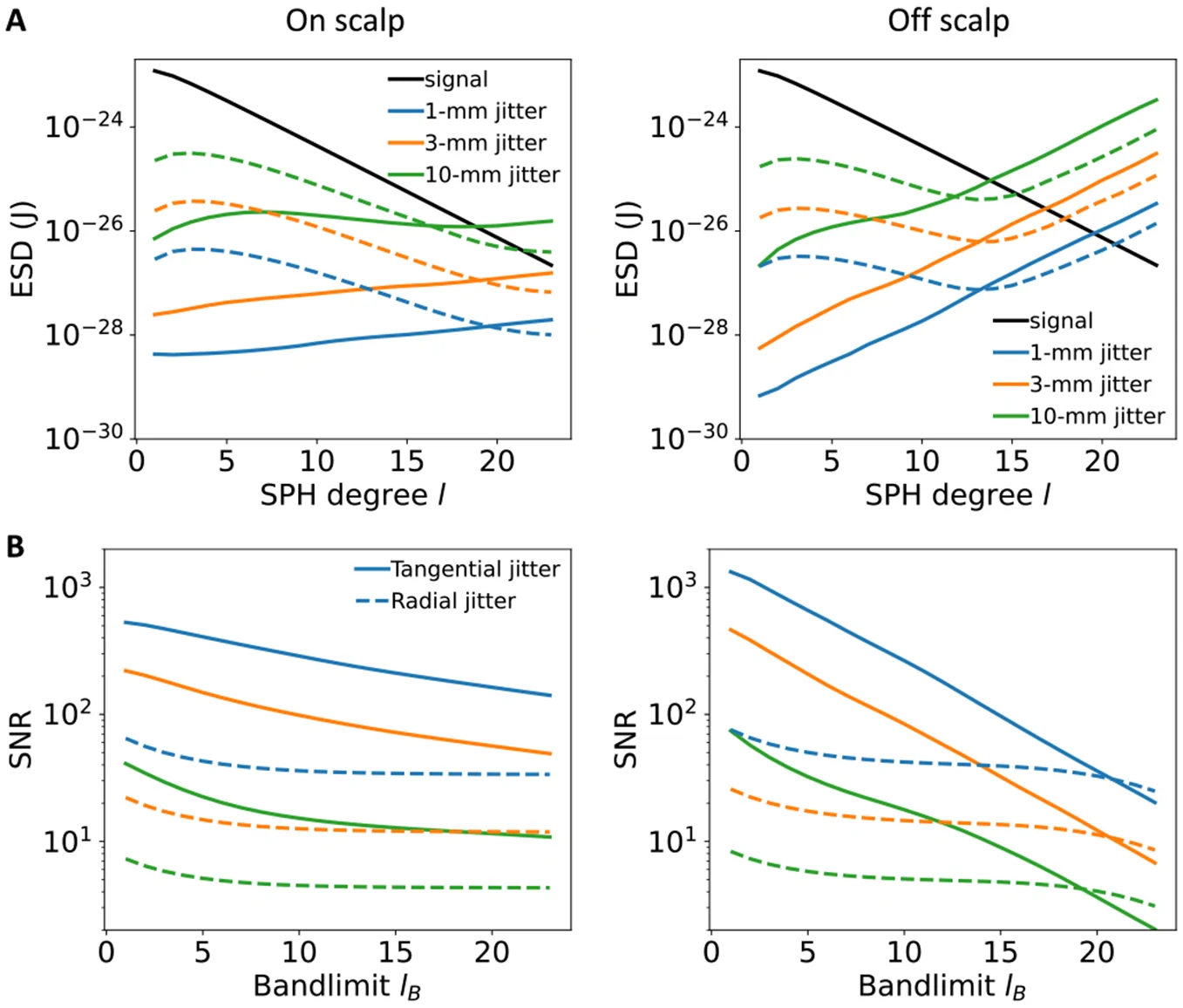
Noise caused by tangential and radial spatial jitter, i.e., random sensor position errors along the tangential and radial directions. **A:** ESDs of the spatial-jitter noise at different levels of jitter for on- and off-scalp measurements of a magnetic field of a tangential current dipole. Solid lines: tangential spatial jitter; dashed lines: radial jitter. The ESD of the magnetic field of the dipole is shown as a black line (the signal). **B:** SNRs of on- and off-scalp measurements as a function of the spherical harmonics bandlimit $l_{B}$ at different levels of tangential and radial spatial jitter.

Fig. 5 shows the ESDs and SNRs due to radial and tangential spatial jitter separately. Radial spatial jitter refers to random sensor displacements along the radial axis, while tangential jitter refers to those in the sensor’s tangential plane. Radial jitter changes the distance to the spherical conductor, while tangential jitter changes it only minimally. The figure shows that radial spatial jitter has a more detrimental effect on the SNR than tangential spatial jitter. At low values of l, the noise due to radial jitter is about two magnitudes larger than that due to tangential jitter. Correspondingly, the SNR with radial jitter is about a factor of ten smaller than that with tangential jitter. The noise due to radial jitter decreases as a function of l, while that due to tangential increases; the noise curves cross at high enough l. At low l, the measurement is limited by radial jitter, while at high l, it is limited by tangential jitter.

At low values of l, radial jitter causes about 35% more noise in on-scalp than in off-scalp measurements. At l≥13, the noise due to radial jitter increases fast as a function of l in off-scalp measurements, while that in on-scalp measurements decreases regardless of the value of l. Depending on the level of radial jitter, the radial-jitter noise becomes higher in off-scalp measurements than in on-scalp measurements when l is larger than 13. Noise due to tangential jitter is initially higher in on-scalp measurements than in off-scalp measurements, but it increases faster as a function of l in off-scalp measurements; the curves cross at l between 6–9 depending on the level of jitter. After that value of l, the tangential-jitter noise is larger in off-scalp than in on-scalp measurements.

**Fig. 6.**
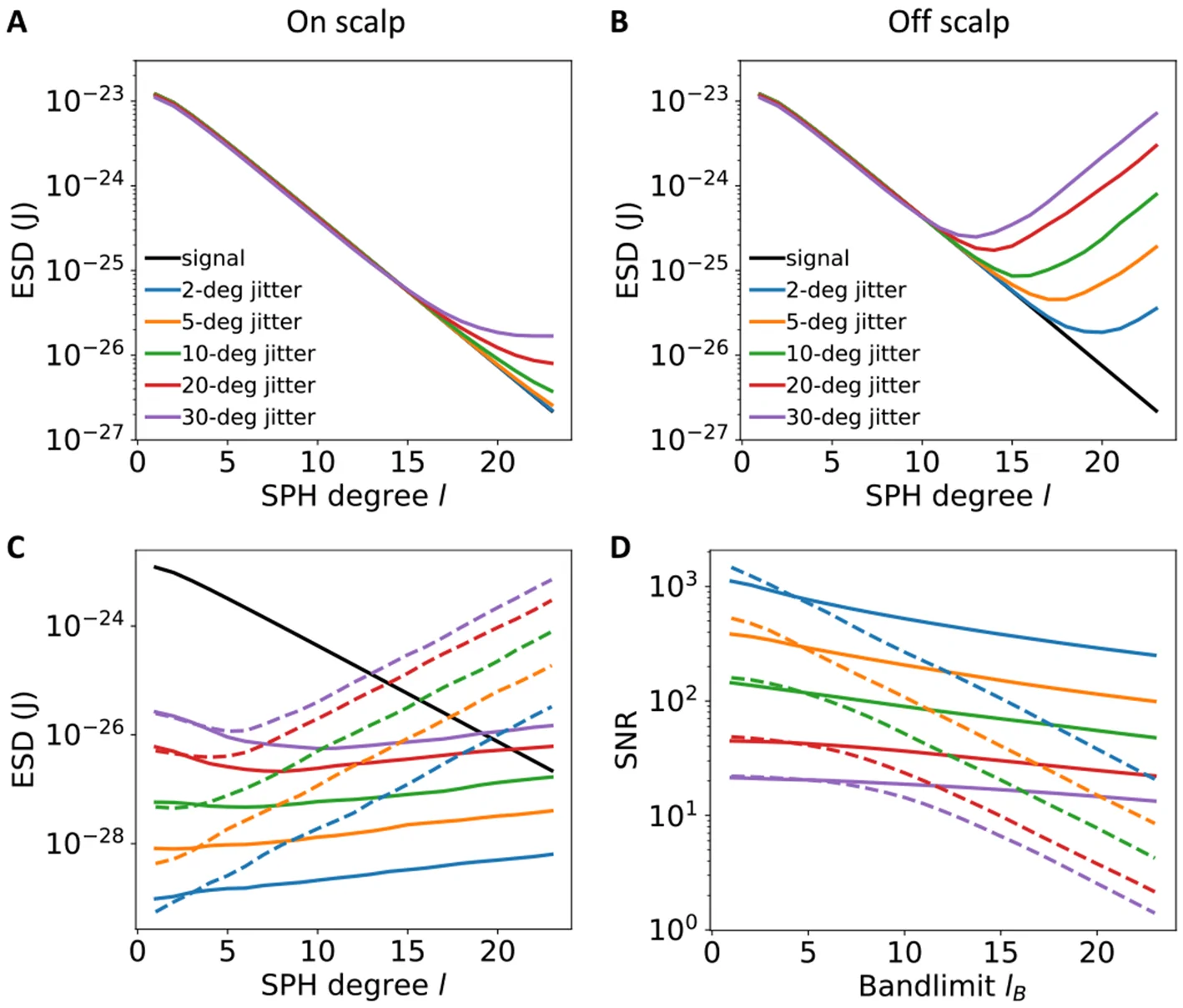
Noise caused by orientation jitter, i.e., random sensor orientation errors. Figure illustration is similar to Fig. 4, please see its caption for panel explanations.

Fig. 6 shows the results for orientation jitter. The results are similar to those for spatial jitter in Fig. 4 but the on- and off-scalp SNRs cross at smaller bandlimits $l_{B}$.

### C. Aperture

**Fig. 7.**
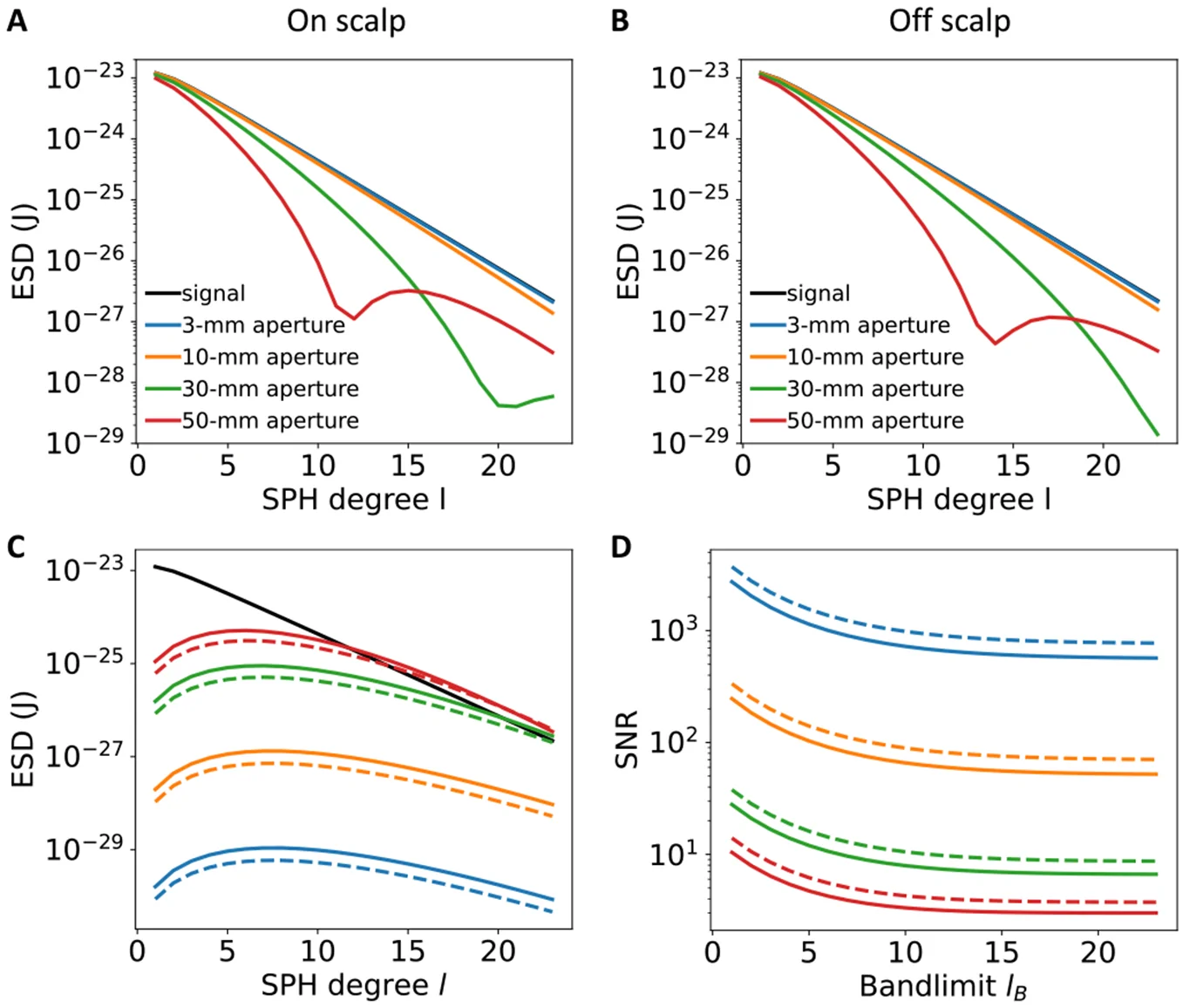
Error caused by sensor aperture, i.e., spatial integration of the magnetic field by the sensor. Figure illustration is similar to Fig. 4, please see its caption for panel explanations.

Fig. 7 shows results for various aperture (sensor) sizes in on- and off-scalp measurements. ESDs reduce more as a function of l as the aperture size increases, i.e., the magnetic field is spatially low-pass filtered by the sensors. At the same aperture size, on-scalp measurements are filtered more than off-scalp measurements. The aperture size has a two-fold effect on the SNR as shown in Fig. 7D. As the aperture size increases, the SNR decreases regardless of the bandlimit $l_{B}$. On the other hand, at the same aperture size, SNR decreases as a function $l_{B}$.

### D. Transfer Functions

**Fig. 8.**
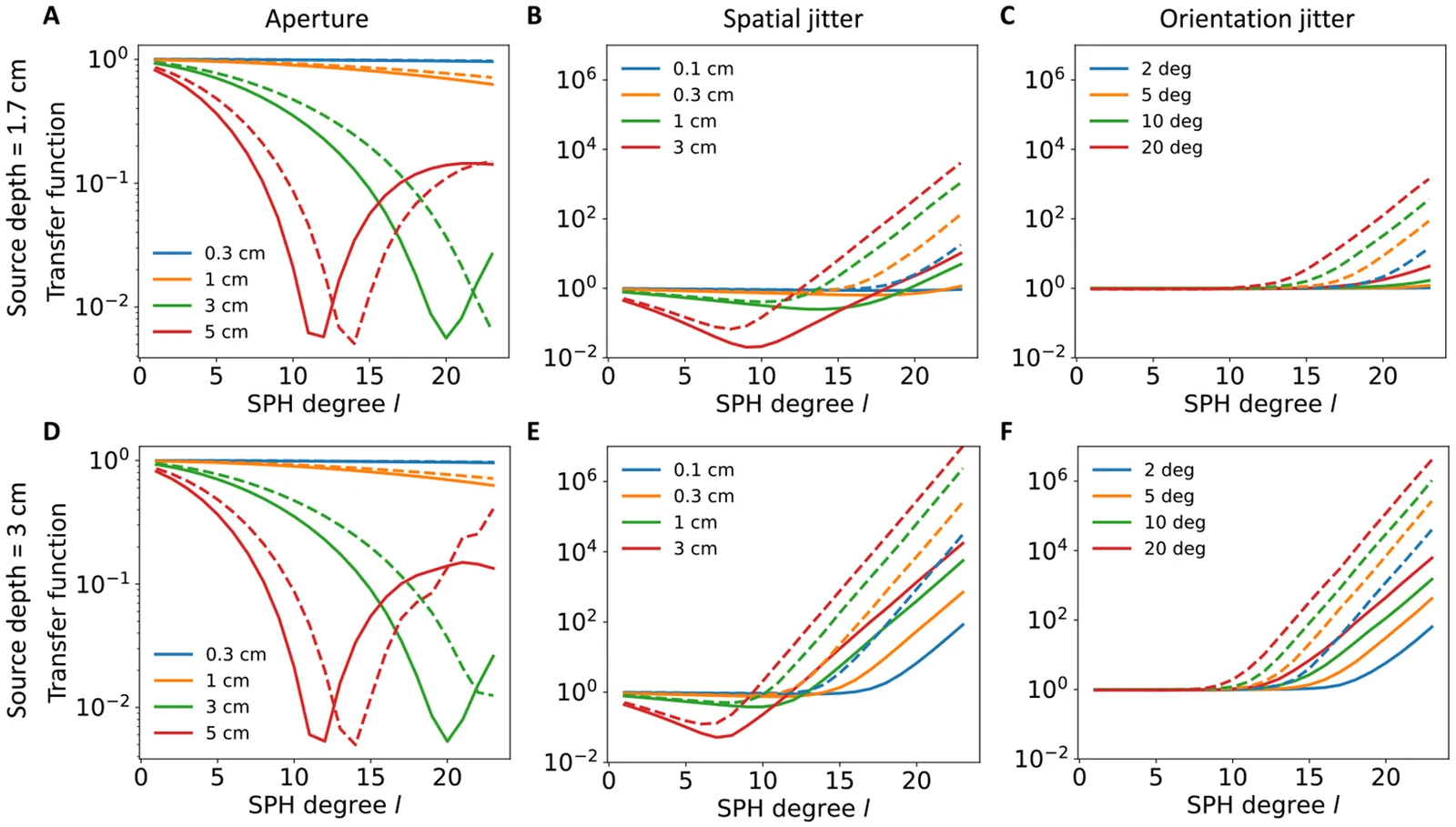
Transfer functions of on-scalp (solid lines) and off-scalp (dashed lines) MEG measurements when they are affected by different levels of aperture error (A; D) as well as spatial (B; E) and orientation jitter (C; F). The transfer functions are shown for magnetic fields from two sources at depths of 1.7 cm (A–C) and 3.0 cm (D–F) from the surface of a spherical conductor. The transfer function is defined as the ratio of the sensor array output and input in the vector spherical harmonics domain.

Fig. 8 summarizes the previous results by showing the transfer functions (Eq. (18)) for aperture, spatial and orientation jitter. The transfer functions are shown for on- and off-scalp field measurements of two sources at depths of 1.7 cm and 3.0 cm from the surface of the spherical conductor.

The figure confirms that on-scalp measurements are spatially low-pass filtered more than off-scalp measurements at the same sensor size. The aperture transfer function does not depend on the source depth. The converse is true for spatial and orientation jitter: off-scalp measurements are affected more than on-scalp measurements at high spatial frequencies at the same level of jitter, and the jitter transfer function depends on the source depth.

### E. Combined Error Sources

Next, the SNR of on-scalp and off-scalp measurements as a function of the source depth is analyzed when the measurements are simultaneously affected by multiple noise and error mechanisms. Here, it is assumed that the on-scalp and off-scalp sensors have rectangular apertures with sidelengths of 3 mm and 25.8 mm, respectively. It is further assumed on-scalp and off-scalp sensor white noise levels of 15 and 3 $\text{fT}∕\sqrt{\text{Hz}}$, respectively. Both the aperture sizes and noise levels are set according to OPM and SQUID sensors [5], [34], [35]. Two scenarios corresponding to different amounts of data averaging are simulated: single-trial data ($N_{\text{trials}}=1$) and an average of 100 individual trials ($N_{\text{trials}}=100$). In the latter case, the sensor white noise level is reduced by a factor of $\sqrt{100}=10$. The measurements are simulated at three levels of jitter (small: 1 mm and 1°; medium: 3 mm and 5°; large: 10 mm and 20°).

**Fig. 9.**
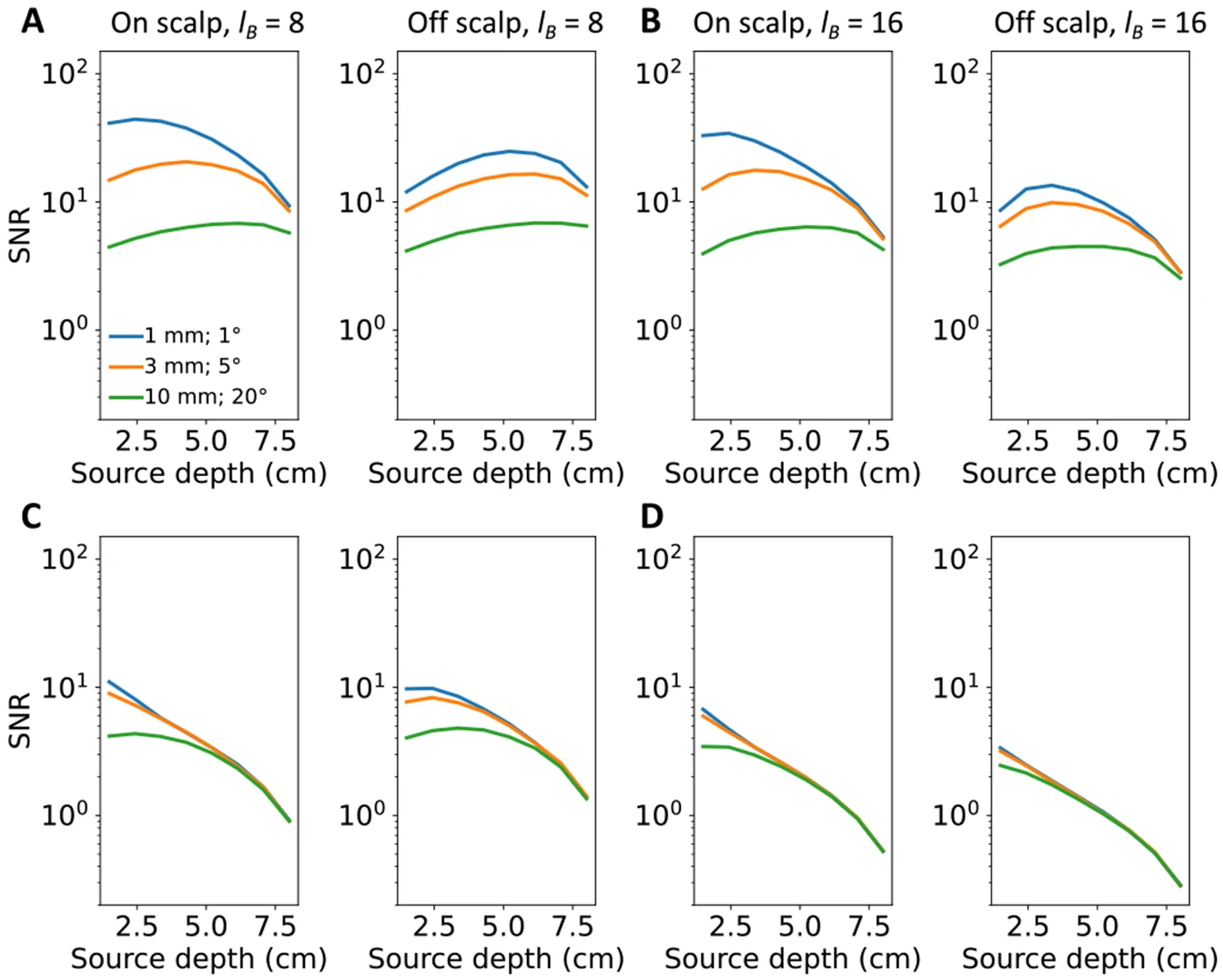
SNRs of on- and off-scalp measurements of magnetic fields due to tangential current dipoles at different depths inside a spherical conductor. The measurements are integrated by the sensor aperture and they are affected by spatial and orientation jitter (small: 1 mm and 1°; medium: 3 mm and 5°; large: 10 mm and 20°). On-scalp (off-scalp) sensors have a rectangular aperture of 3 mm (25.8 mm) and sensor noise of 15 $\text{fT}∕\sqrt{\text{Hz}}\;(3\;\text{fT}∕\sqrt{\text{Hz}})$. The data are either single trial (N=1) or averaged over 100 trials (N=100). A: $l_{B}=8$; N=100. B: $l_{B}=16$; N=100. C: $l_{B}=8$; N=1. D: $l_{B}=16$; N=1.

Fig. 9 shows the results of this analysis at two bandlimits ($l_{B}=8$ and $l_{B}=16$). With single-trial data ($N_{\text{trials}}=1$), small-to-medium jitter has negligible effect on the SNR; the SNR is mainly limited by sensor white noise. However, large jitter still reduces the SNR in this case, especially for the superficial sources. At $l_{B}=8$ and at the same level of spatial jitter, off-scalp SNR is higher than on-scalp SNR at all source depths except for the most superficial source; at $l_{B}=16$, it is the other way around, on-scalp SNR is higher.

With lower sensor noise level ($N_{\text{trials}}=100$), spatial jitter has a more severe effect on the SNR. Interestingly, jitter causes the source depth–SNR curves to flatten. Some of the curves even have a reverse U-shape. For example, small-jitter off-scalp SNR at $l_{B}=8$ peaks for a source at a depth of 5-cm (SNR = 25), while it is the lowest for the most superficial and deepest sources (SNR ~ 12). At $l_{B}=16$, the on-scalp SNR is higher than off-scalp SNR at the same level of spatial jitter. At $l_{B}=8$ and at the same level of spatial jitter, the on-scalp SNR is higher for the superficial sources, but the off-scalp SNR is higher for sources deeper than about 6 cm.

### F. Common Translation and Rotation

**Fig. 10.**
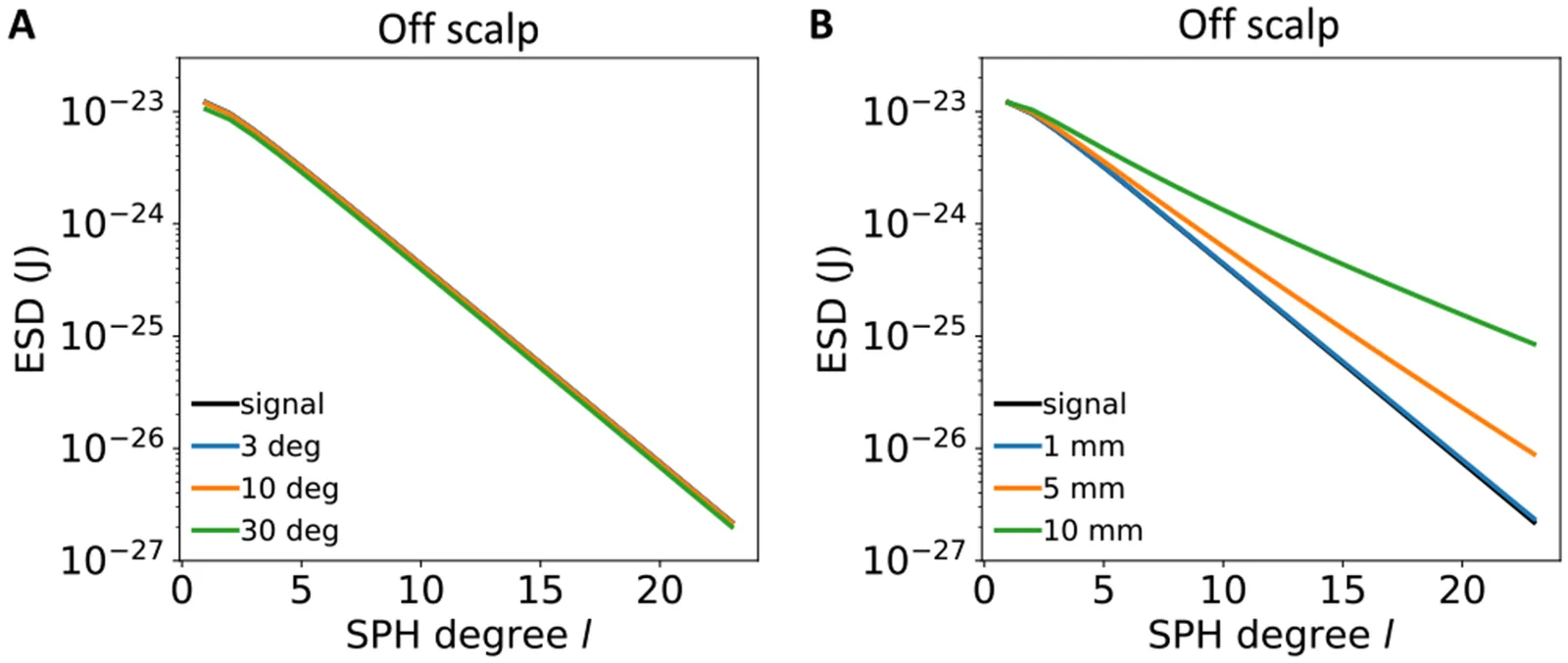
Noise caused by uniform random rotations and translations of the whole sensor array. **A:** ESD of the off-scalp measurement of a magnetic field of a tangential current dipole at different levels of random rotations of the whole measurement array. **B:** As in A, but for random translations.

Fig. 10 shows the ESDs of the off-scalp measurements when the measurement sensor array is affected by either uniform random rotations or translations. The results are only shown for off-scalp measurements as they are the same for on-scalp measurements, i.e., uniform rotations and translations affect them the same way. Fig. 10A shows that random rotations of the whole sensor array do not have an effect on the resulting ESD, i.e., they do not change the magnetic energy. Fig. 10B illustrates that random translations cause l-dependent noise on the ESD: they mainly affect the slope how the ESD scales as a function of l.

### G. Realistic Geometry

**Fig. 11.**
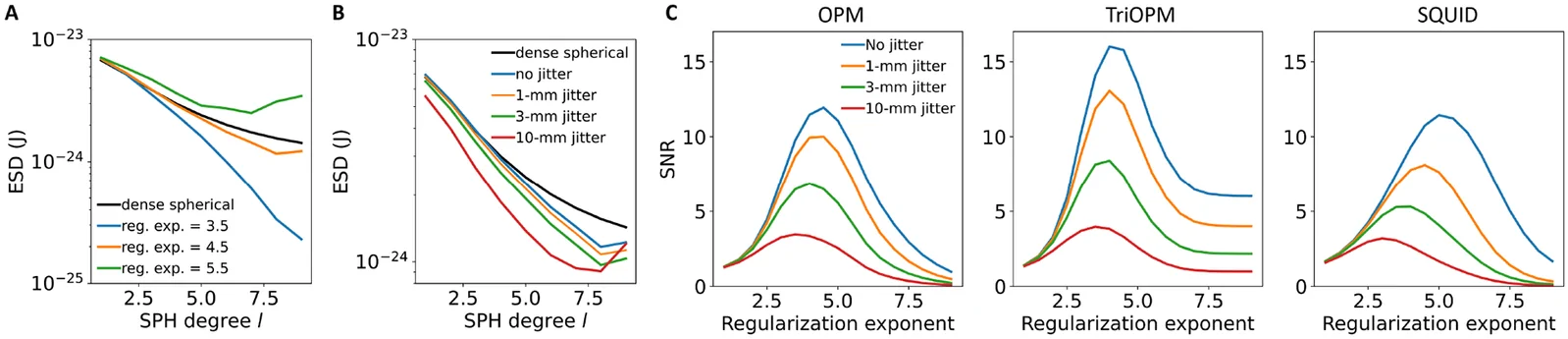
Effects of spatial jitter on magnetic-field measurements with realistic sensor arrays in a realistic geometry. **A:** Energy-spectral densities (ESDs) of magnetic fields of current dipoles estimated from measurements with a 102-sensor single-axis OPM array. Different regularization exponents in the least-squares method were used to estimate the vector-spherical-harmonics (VSH) coefficients. ESD obtained with a dense spherical array of triaxial point-like magnetometers is also shown. The ESDs are averaged over all sources in the cortex. **B:** ESDs, as averaged over the sources, estimated with 102 single-axis OPM measurements affected with different amounts of spatial jitter. Regularization exponent of 4.5 was used. **C:** Signal-to-noise ratios (SNRs) of the VSH reconstructions at a bandlimit of $l_{B}=9$ as a function of the regularization exponent for measurements affected with different amounts of spatial jitter. The SNRs are averaged across the sources and are shown for three sensor arrays: 102-sensor single-axis OPM array, 102-sensor (or 306-channel) triaxial TriOPM array and 102-sensor SQUID magnetometer array.

Fig. 11 shows the simulation results for realistic sensor arrays in a realistic geometry. Fig. 11A shows the effect of the regularization parameter in the least-squares operator (Eq. (21)) on the estimated ESD as averaged over the cortical sources. With a regularization exponent of 4.5, the estimated average ESD for the single-axis OPM array is closest to that obtained with the dense spherical array. Regularization exponents of 3.5 and 5.5 either under- or overestimate the ESD values, respectively, reducing the accuracy of the VSH coefficients. Fig. 11B shows the effect of spatial jitter on the ESDs estimated with the OPM sensor array. Spatial jitter of the OPM sensor positions causes noise on the estimated VSH coefficients, which shows up as a reduction in the estimated ESD values.

Fig. 11C shows the average SNRs of the VSH coefficient reconstructions of 102-sensor single-axis OPM, 102-sensor triaxial TriOPM (306 channels) and 102-sensor SQUID magnetometer arrays as a function of the regularization exponent, when the sensor positions are affected by different amounts of spatial jitter. When the measurements are affected by spatial jitter, two effects on the SNR can be seen. First, as spatial jitter increases, the SNR that can be achieved decreases. Second, as jitter increases, the regularization exponent that yields the highest SNR is reduced. TriOPM yields the highest SNR values, while SQUID yields the lowest. For example, with a spatial jitter of 1 mm, average SNRs of 10.0, 13.1 and 8.1 are achieved with OPM, TriOPM and SQUID, with regularization exponents of 4.5, 4.0 and 4.5, respectively. With a jitter of 10 mm, the corresponding SNRs are reduced to 3.5, 4.0 and 3.2, which are achieved with regularization exponents of 3.5, 3.5 and 3.0, respectively.

**Fig. 12.**
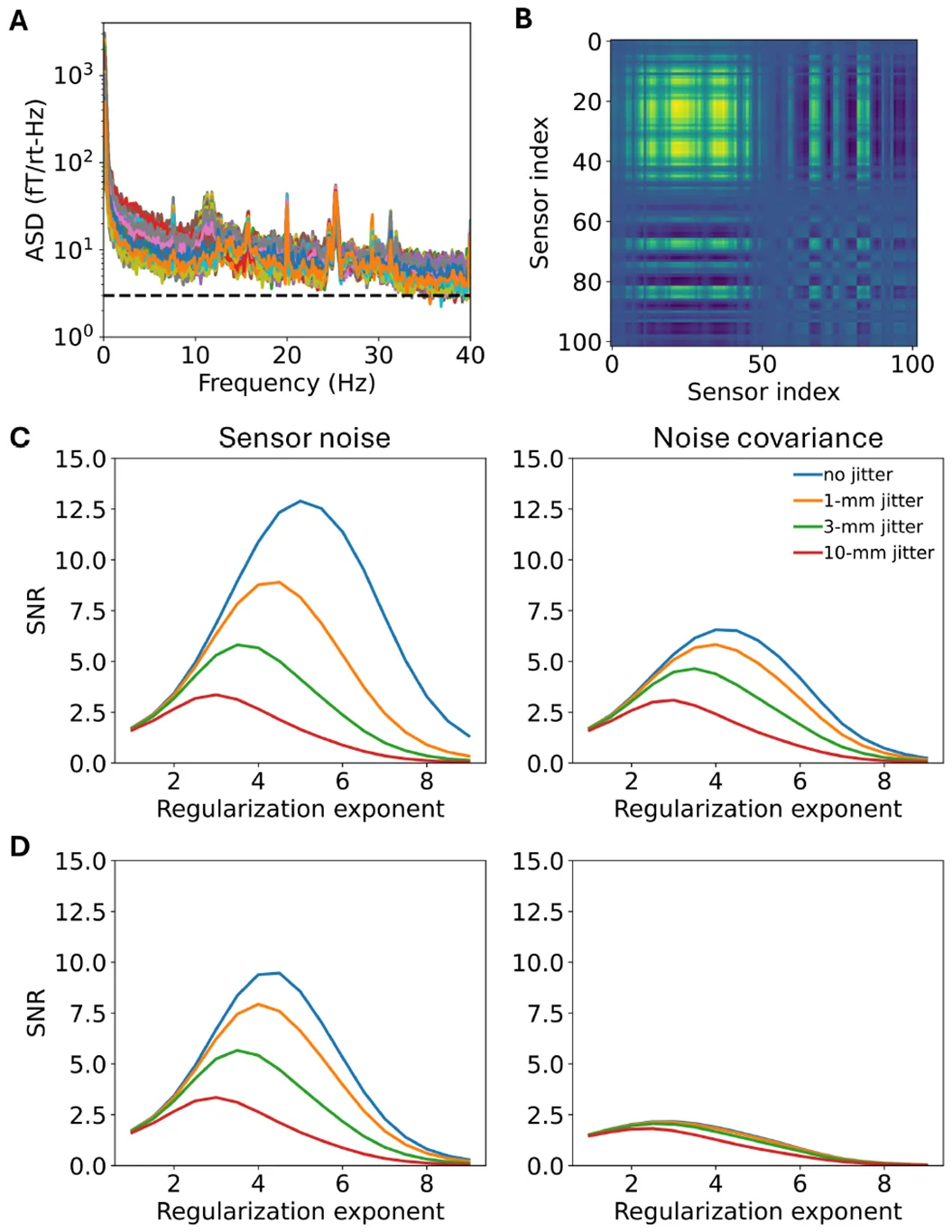
Spatial-jitter simulations with a 102-sensor SQUID magnetometer array in a realistic geometry with simulated sensor noise and empty-room noise covariance matrix. **A:** The measured amplitude-spectral densities (ASDs) of the SQUID sensors in the empty-room condition. The dashed black line illustrates the sensor white noise level of 3 $\text{fT}∕\sqrt{\text{Hz}}$ used also in the simulations. **B:** The noise covariance matrix of the empty-room recording. **C:** The SNR of the VSH reconstruction at a bandlimit of $l_{B}=9$ as a function of the regularization exponent for different levels of spatial jitter. Here, either sensor noise (left) or noise generated using the estimated noise covariance (right) is included in the simulation. The plots are similar to those shown in Fig. 11C. **D:** The SNR as in panel C but for a case where the source amplitude is reduced by a factor of ten.

Fig. 12 shows the simulation results for the SQUID sensor array using the two noise models. Fig. 12A illustrates the empty-room noise spectral densities across the sensors, while Fig. 12B shows the empty-room noise covariance matrix. Fig. 12C shows the SNR as averaged over the sources as a function of the regularization exponent for different levels of spatial jitter and the two noise models. Spatial jitter reduces SNR and changes the regularization exponent which gives the maximum SNR in both noise models. SNR is higher with the sensor noise model than with the covariance model. Fig. 12D shows the SNRs when the source amplitude is reduced by a factor of ten. In this case, spatial jitter does not have a considerable effect on the SNR obtained with the noise covariance model. These results show that spatial jitter limits the SNR in cases where the sensor and environmental noise is small compared to the signal.

## V. Discussion

This paper presented a theoretical framework to assess random sensor position errors in MEG. The paper argues that random sensor position errors affect the spatial sampling of the quasistatic magnetic field similarly as jitter affects temporal signal acquisition. In addition to random sensor position errors (‘spatial jitter’), the paper considered other noise sources such as random sensor orientation errors (‘orientation jitter’) and errors due to sensor dimensions (‘aperture errors’).

Spatial and orientation jitter in real sensor arrays may arise from multiple sources. They could be due to errors in the geometrical model of the sensor and the sensor array. This type of a sensor position error affects mostly coregistration methods, where the sensor positions are obtained by aligning a CAD model of the sensor helmet with an optical scan of the participant [36]. The errors in the geometrical model may be due to, e.g., errors in the position of the vapor cell in the OPM sensor housing, errors in the direction of the laser beam in the OPM or other errors and tolerances in the geometrical models. If the sensors were localized using magnetic fields, the sensor position errors would also be partly random and independent across sensors as the method localizes each sensor individually and random errors of the method propagate to each sensor individually [37].

In the heart of the analogy between temporal signal sampling and spatial field sampling is signal representation with a basis that consists of orthogonal functions with increasing frequency. In one dimension, a convenient choice is the Fourier basis, while for the quasistatic neuromagnetic field it is the vector spherical harmonic (VSH) representation. Parseval’s theorem stating that the signal energy is the same in temporal (spatial) and frequency (spatial frequency) domains equally applies for both temporal and spatial neuromagnetic field signals.

Here, the VSH functions were normalized so that each of them carries one unit of magnetic energy. This simplifies the form of the Parseval’s theorem (Eq. (12)), but it also has important implications. Using this normalization, magnetic sensor arrays at different distances from the source recover the same VSH coefficients (Fig. 2A). This means that even though arrays at different distances from the scalp yield different measurements, they effectively recover the same magnetic field; using the VSH coefficients the field can be extrapolated anywhere. So the on-scalp magnetic field can be extrapolated from the off-scalp measurement using the same coefficients and vice versa. However, as the sensor-to-scalp distance increases, spatial white (sensor) noise maps more strongly to the VSH coefficients (same level of noise gives larger coefficients), reducing the SNR of the measurement. This effect was spatial-frequency dependent and led to a steeper increase in the noise as a function of l when the sensor-to-scalp distance increased. This effect was also seen with spatial and orientation jitter: the same level of jitter had a stronger effect in off-scalp measurements than in on-scalp measurements at high spatial frequencies. Thus, as the sensor-to-scalp distance increases the measurement becomes more sensitive to noise, limiting SNR and the spatial resolution of the measurement and reducing the field extrapolation accuracy. Thereby, it may be easier to reach higher spatial resolution with measurements that are closer to the scalp than with those that are further away even if the closer measurements have larger sensor position and orientation errors and higher level of sensor noise.

Radial spatial jitter (displacing sensors so that they move further from the head) had a more detrimental effect on the SNR and caused more noise at spatial frequencies l<15 than tangential spatial jitter, that maintains the sensor-to-head distance. At low spatial frequencies (l<5), radial jitter caused about a hundred times more noise than tangential jitter. Radial spatial jitter also caused more noise in on-scalp than in off-scalp measurements at spatial frequencies l<13. From the practical perspective, low spatial frequencies have the highest SNR while being the most affected by radial jitter. It is, therefore, important to minimize the sensor-position uncertainty especially along the radial direction.

Aperture errors behaved differently than jitter noise: the same aperture size caused more error in on-scalp than in off-scalp measurements at all spatial frequencies. This is because on-scalp measurements are more prone to spatial filtering than off-scalp measurements due to the closer distance to the scalp. Generally, having a sensor with as small dimensions as possible seems favorable as the sensor dimensions will always cause errors and low-pass filter VSH components. However, this is a more nuanced question than the previous sentence anticipates, and it needs more investigation due to two reasons. First, the spatial low-pass filtering by the sensor can act as a spatial anti-alias filter and reduce the aliasing of high-order VSH components if sufficient number of sensors is not used or the sensors do not have an adequate coverage. Second, sensor noise in both OPMs and SQUIDs scales as a function of the sensor dimension [38], [39].

The combined effects of spatial jitter and aperture errors on the SNR are quite complex as shown in Fig. 9. While usually (as in the case with sensor white noise; Fig. 3), the measurement SNR decreases as a function of source depth, here, spatial jitter and aperture errors can lead to a situation where the SNR–source-depth curve is relatively flat or even reverse U-shaped. This is because the SNR of a superficial source may be affected more by jitter than that from a deeper source (Fig. 8). The jitter noise is conditional on the signal; this can lead to a situation where the jitter noise reduces faster as a function of the source depth than the signal energy leading to an increase in the SNR.

Random rotations of the whole sensor array did not change the l-dependency of the ESD, but scrambled its dependency on the m-order. This is because random rotations do not change the shape of the magnetic field, but only rotate the field pattern as seen by the sensor array to a different angle. On the other hand, from the reference frame of the sensor array, random rotations change the orientation of the source. Hence, random rotations do not affect the magnetic energy and its distribution. However, in real-world sensor arrays, rotations may lose magnetic energy as the field pattern may be rotated so that it is not covered by the sensor array. Random translations of the whole sensor array caused l-dependent noise. This was mainly manifested as a change in the slope of how the ESD scales as a function of l. Random translations change the distance of the sensor array to the source, and, thereby, the magnetic energy is changed. Random rotations and translations of the whole sensor array affected on- and off-scalp measurements similarly. The suggested reason for this is as follows. Random translations change the sensor-to-source distance. As on- and off-scalp measurements yield the same VSH coefficients for the original and translated source, the error is also the same for both measurements.

Spatial jitter reduced the SNR of realistic sensor arrays and changed the regularization exponent of the least-squares operator that yielded the least noise in the VSH-coefficient estimation. Thereby, in addition to providing a theoretical and simulation tool to facilitate understanding of sensor position and aperture errors on MEG measurements, the spatial-jitter model could be also applied in analysis and modeling of realistic MEG measurements. The model could be used to inform the choice of regularization parameter in the inverse operator given the estimated sensor position error and sensor size. Additionally, the model itself could be used to construct the regularization operator. For example, the minimum-normestimate (MNE) inverse operator includes a noise-covariance matrix that represents the estimated statistics of the measurement noise [40], [41], [42]. Effectively, the noise-covariance matrix regularizes the MNE inverse operator. The noise-covariance matrix is typically estimated from data but, to this end, a component that represents the expected spatial-jitter noise and aperture error calculated based on the estimated sensor position error and sensor size could be added into it.

The energy normalization of the VSHs is theoretically elegant, but it also has practical value. It could be used to normalize the VSHs in the signal-space separation (SSS) method applied mostly in SQUID-MEG [24]. In contrast to any numerical or mathematical normalization scheme, it would provide a physics-based normalization bringing the VSHs with different degrees to common units. To this end, also the external VSHs representing interference could be normalized similarly (Appendix A). Similar analysis as conducted here could be applied to inform the choice of the suitable expansion order for the SSS method given the expected sensor position errors, or the SSS operator could be regularized against these errors.

Previous publications have shown that OPMs can yield higher signal amplitude, information capacity and higher spatial frequencies than SQUID-based MEG [5], [6], [17]. Intuitively, this would suggest that OPM sensor positions should be known more accurately than SQUID positions. However, this is a complicated question that depends on many factors and parameters such as assumed sensor noise level, SNR and number of sensors. While the effect of the previous parameters has been modeled in the previous studies, other parameters have not been taken into account such as the regularization of the matrix inversion and the spatial bandlimit of the measurement ($l_{B}$). In relation to the previous studies, the result of this paper can be summarized as follows. In the limit of dense spatial sampling, on-scalp MEG should have easier access to high spatial frequencies than off-scalp MEG, but at the same time at low spatial frequencies on-scalp MEG may be affected more by spatial jitter (Fig. 4). In the case of sensor-noise-limited SNR and dense sampling (Fig. 9C-D), at a high bandlimit $l_{B}=16$ on-scalp MEG gives better SNR than off-scalp MEG at all levels of spatial jitter; at a lower bandlimit $l_{B}=8$, off-scalp MEG gives better SNR than on-scalp MEG. In the spatial-jitter-limited case and dense sampling (Fig. 9A-B), the on-scalp SNR is higher than that of off-scalp at $l_{B}=16$ at all levels of spatial jitter. On the other hand, on-scalp and off-scalp MEG compete at $l_{B}=8$: the on-scalp SNR is higher for the superficial sources, but the off-scalp SNR is higher for sources deeper than about 6 cm. For realistic sensor arrays at a bandlimit of $l_{B}=9$, OPMs yield higher SNR values than SQUIDs at all levels of spatial jitter, while triaxial OPMs give higher SNR than single-axis OPMs. This indicates the benefit of sensing the full magnetic field vector: triaxial OPMs with a 1-mm of spatial jitter can still yield SNR as high as jitterless single-axis OPM array. Altogether, the results indicate that OPMs have better access to high spatial frequencies than SQUIDs, and that realistic OPM arrays give higher SNRs than SQUIDs at high bandlimits, and are more resistant to spatial jitter, especially triaxial OPMs.

## VI. Limitations

The analysis presented in this manuscript was conducted to introduce and validate the spatial-jitter model and to investigate its effects at different distances from the sources. Even though some of the simulations were modeled according to real OPM and SQUID sensors, the results are not necessarily intended to assess the real-world performance of OPM- and SQUID-based sensor arrays. Drawing practical implications based on the simulation results is complicated by the fact that sensor position error mechanisms are generally different in OPM and SQUID arrays. OPMs allow for individual positioning and may have errors due to the sensor CAD model tolerances and OPM physics as outlined previously. Thereby, they exhibit sensor-wise random position errors. The sensor array geometry in SQUID-based MEG arrays is usually very well known and the sensor position errors mainly manifest as systematic translations and rotations of the whole sensor array with respect to the subject’s head. However, small random position and orientation errors may still exist, whose effects on the SSS performance of SQUID-sensor arrays have been investigated [43].

While vector spherical harmonics allow for analytical calculations and manipulation, the results shown in this paper were based on simulations. Analytical formulas that describe the spatial-jitter phenomena on the VSH domain could be useful, for example, in rapid numerical construction of the jitter-noise-covariance matrix.

The spatial-jitter model was demonstrated using simulations. The model could also be tested experimentally if enough OPM sensors were available for robust estimation of the VSH coefficients. For example, the magnetic field from a spherical current-dipole phantom could be measured with an OPM array. Random sensor-wise displacements could be added to the OPM sensor positions and the field would be measured again. Then, the VSH coefficients would be estimated from the measurements with the jittered sensor positions with the assumption that the sensors were in their original positions. These coefficients would be then compared against those obtained without spatial jitter.

The proposed spatial-jitter model was based on the VSH representation of the magnetic field and, thereby, it applies in the proposed form for cases where the VSH representation is valid. Shortly, the VSH representation converges at all points that are outside a sphere that encapsulates all the magnetic field sources. This is typically valid for SQUID-based MEG measurements, but is not always valid for OPM-based measurements. In the latter case, a sphere that covers all the sources and does not intersect with the sensor array cannot be always fit. However, to address this issue, solutions have been proposed such as the multi-origin SSS and the prolate spheroidal harmonics [44], [45]. To this end, the spatial-jitter model could be possibly incorporated with these methods for more accurate modeling for OPMs.

The single-axis and triaxial OPM sensors were modeled by uniformly integrating the magnetic field within a 3 × 3 × 3 mm^3^ vapor cell. The dimensions of the vapor cell were set as in the Gen-1 and Gen-3 OPMs of QuSpin Inc. However, this sensor model does not necessarily represent the behavior of a real OPM sensor, in which the integration volume is defined by physical parameters such as the laser-beam dimensions, attenuation of the laser beam in the atomic vapor and atomic diffusion in the cell. In a real OPM sensor, the integration volume is most likely smaller than the whole vapor cell. Additionally, in the triaxial QuSpin OPM, the different field components are measured at slightly different positions in the vapor cell [46]. However, the error due to assuming a 3-mm vs. point-like aperture should be small when l<23 (Fig. 7A).

## VII. Conclusion

This paper developed a spatial-jitter model for spatial sampling of the quasistatic magnetic field. The model can be used to analyze the effect of sensor position, orientation and dimension errors on the achievable SNR and spatial resolution in MEG. Similarly to temporal jitter, spatial jitter causes (spatial-)frequency-dependent noise that limits the acquisition SNR. The spatial-jitter model could be used in MEG data modeling to improve, for example, source estimation.

## Appendix A

### Appendix A: Energy Normalization of Vector Spherical Harmonics

The inner field components are defined as

(22)
$$\vec{B}(\vec{r})=-\mu_{0}\sum_{l=1}^{\infty }\sum_{m=-l}^{l}\alpha_{lm}\sqrt{\left(l+1)(2l+1\right)}\frac{{\vec{V}}_{lm}(\theta ,\phi )}{r^{l+2}},$$

and the magnetic energy outside the source sphere is

(23)
$$E=\frac{1}{2}\int_{r>R}\frac{B^{2}}{\mu_{0}}dV=\frac{1}{2}\int_{R}^{\infty }\frac{\vec{B}\cdot \vec{B}}{\mu_{0}}r^{2}d\Omega dr.$$

Using the orthogonality relation of VSHs (Eq. (10)), simplifies the magnetic energy into

(24)
$$E=\frac{1}{2}\sum_{l=1}^{\infty }\sum_{m=-l}^{l}\mu_{0}{|\alpha_{lm}|}^{2}(l+1)(2l+1)\int_{R}^{\infty }\frac{1}{r^{2l+4}}r^{2}dr\;\;=\frac{1}{2}\sum_{l=1}^{\infty }\sum_{m=-l}^{l}\mu_{0}{|\alpha_{lm}|}^{2}\frac{\left(l+1)(2l+1\right)}{2l+1}\frac{1}{R^{2l+1}}\;\;=\frac{1}{2}\sum_{l=1}^{\infty }\sum_{m=-l}^{l}\mu_{0}{|\alpha_{lm}|}^{2}\frac{\left(l+1\right)}{R^{2l+1}}.$$

Thereby, we get the magnetic-field basis vectors in Eq. (11) that are normalized to one unit of magnetic energy and simplify the magnetic energy formula (Eq. (12)) by defining

(25)
$$a_{lm}=\sqrt{\mu_{0}\frac{\left(l+1\right)}{R^{2l+1}}}\alpha_{lm},$$

and

(26)
$${\vec{b}}_{lm}(\vec{r})=-\sqrt{\mu_{0}R^{2l+1}(2l+1)}\frac{{\vec{V}}_{lm}(\theta ,\phi )}{r^{l+2}}.$$

For completeness, the result is also shown for the external field components, which are defined as

(27)
$$\vec{B}(\vec{r})=-\mu_{0}\sum_{l=1}^{\infty }\sum_{m=-l}^{l}\gamma_{lm}\sqrt{l(2l+1)}r^{l-1}{\vec{W}}_{lm}(\theta ,\phi ).$$

This time the magnetic energy inside the sphere is

(28)
$$E=\frac{1}{2}\sum_{l=1}^{\infty }\sum_{m=-l}^{l}\mu_{0}{|\gamma_{lm}|}^{2}lR^{2l+1},$$

and the normalized basis functions ${\vec{w}}_{lm}(\vec{r})$ with coefficients $g_{m}$ are obtained by defining

(29)
$$g_{lm}=\sqrt{\mu_{0}lR^{2l+1}}\gamma_{lm},$$

and

(30)
$${\vec{w}}_{lm}(\vec{r})=-\sqrt{\mu_{0}\frac{\left(2l+1\right)}{R^{2l+1}}}r^{l-1}{\vec{W}}_{lm}(\theta ,\phi ).$$

## References

[1] Hämäläinen M, Hari R, Ilmoniemi RJ, Knuutila J, and Lounasmaa OV, “Magnetoencephalography—Theory, instrumentation, and applications to noninvasive studies of the working human brain,” Rev. Mod. Phys, vol. 65, no. 2, pp. 413–497, Apr. 1993.

[2] Baillet S, “Magnetoencephalography for brain electrophysiology and imaging,” Nature Neurosci, vol. 20, no. 3, pp. 327–339, Mar. 2017. 28230841 10.1038/nn.4504

[3] Budker D and Romalis M, “Optical magnetometry,” Nature Phys, vol. 3, no. 4, pp. 227–234, 2007.

[4] Boto E , “On the potential of a new generation of magnetometers for MEG: A beamformer simulation study,” PLoS ONE, vol. 11, no. 8, Aug. 2016, Art. no. e0157655. 27564416 10.1371/journal.pone.0157655 PMC5001648

[5] Iivanainen J, Stenroos M, and Parkkonen L, “Measuring MEG closer to the brain: Performance of on-scalp sensor arrays,” NeuroImage, vol. 147, pp. 542–553, Feb. 2017. 28007515 10.1016/j.neuroimage.2016.12.048 PMC5432137

[6] Iivanainen J, Mäkinen AJ, Zetter R, Stenroos M, Ilmoniemi RJ, and Parkkonen L, “Spatial sampling of MEG and EEG based on generalized spatial-frequency analysis and optimal design,” NeuroImage, vol. 245, Dec. 2021, Art. no. 118747. 34852277 10.1016/j.neuroimage.2021.118747 PMC8752968

[7] Wens V, “Exploring the limits of MEG spatial resolution with multipolar expansions,” NeuroImage, vol. 270, Apr. 2023, Art. no. 119953. 10.1016/j.neuroimage.2023.119953 36842521

[8] Brookes MJ , “Theoretical advantages of a triaxial optically pumped magnetometer magnetoencephalography system,” NeuroImage, vol. 236, Aug. 2021, Art. no. 118025. 33838266 10.1016/j.neuroimage.2021.118025 PMC8249355

[9] Schoffelen JM, Cheung T, Knappe S, and Oostenveld R, “Optimal configuration of on-scalp OPMs with fixed channel counts,” Imag. Neurosci, vol. 3, May 2025, Art. no. IMAG.a.22. 10.1162/IMAG.a.22 PMC12320008 40800964

[10] Tierney TM , “Pragmatic spatial sampling for wearable MEG arrays,” Sci. Rep, vol. 10, no. 1, p. 21609, Dec. 2020. 33303793 10.1038/s41598-020-77589-8 PMC7729945

[11] Beltrachini L, von Ellenrieder N, Eichardt R, and Haueisen J, “Optimal design of on-scalp electromagnetic sensor arrays for brain source localisation,” Human Brain Mapping, vol. 42, no. 15, pp. 4869–4879, Oct. 2021. 34245061 10.1002/hbm.25586 PMC8449117

[12] Takeda Y , “Sensor array design of optically pumped magnetometers for accurately estimating source currents,” NeuroImage, vol. 277, Aug. 2023, Art. no. 120257. 37392806 10.1016/j.neuroimage.2023.120257

[13] Zhdanov A, Nurminen J, Iivanainen J, and Taulu S, “A minimum assumption approach to MEG sensor array design,” Phys. Med. Biol, vol. 68, no. 17, Sep. 2023, Art. no. 175030. 10.1088/1361-6560/ace306 PMC10481949 37385260

[14] Wang W , “Design of locally arranged sensor arrays in wearable OPM-MEG based on sensor volume constraints,” Measurement, vol. 229, Apr. 2024, Art. no. 114373.

[15] Marhl U, Hren R, Sander T, and Jazbinšek V, “Optimization of OPM-MEG layouts with a limited number of sensors,” Sensors, vol. 25, no. 9, p. 2706, Apr. 2025. 40363144 10.3390/s25092706 PMC12074169

[16] Yeo W-J, Taulu S, and Nathan Kutz J, “Effcient magnetometer sensor array selection for signal reconstruction and brain source localization,” 2022, arXiv:2205.10925.

[17] Zetter R, Iivanainen J, Stenroos M, and Parkkonen L, “Requirements for coregistration accuracy in on-scalp MEG,” Brain Topography, vol. 31, no. 6, pp. 931–948, Nov. 2018. 29934728 10.1007/s10548-018-0656-5 PMC6182446

[18] Qi S , “Evaluating the effect of OPM array errors on the multiple dipole localization accuracy of OPM-MEG,” Measurement, vol. 254, Oct. 2025, Art. no. 117764.

[19] Nugent AC, Benitez Andonegui A, Holroyd T, and Robinson SE, “On-scalp magnetocorticography with optically pumped magnetometers: Simulated performance in resolving simultaneous sources,” Neuroimage, Rep, vol. 2, no. 2, Jun. 2022, Art. no. 100093. 35692456 10.1016/j.ynirp.2022.100093 PMC9186482

[20] Pang M, Huang Z, Ding Z, and Han B, “Co-registration error analysis and array calibration for OPM-MEG system,” IEEE Trans. Instrum. Meas, vol. 71, pp. 1–12, 2022.

[21] Kobayashi H , “Sampling jitter and finite aperture time effects in wideband data acquisition systems,” IEICE Trans. Fundamentals Electron, pp. 335–346, 2002.

[22] Cohen L, Time-frequency Analysis, vol. 778. Upper Saddle River, NJ, USA: Prentice-Hall, 1995.

[23] Jackson JD, Classical Electrodynamics. Hoboken, NJ, USA: Wiley, 2021.

[24] Taulu S and Kajola M, “Presentation of electromagnetic multichannel data: The signal space separation method,” J. Appl. Phys, vol. 97, no. 12, Jun. 2005, Art. no. 124905.

[25] Mäkinen AJ, Zetter R, Iivanainen J, Zevenhoven KCJ, Parkkonen L, and Ilmoniemi RJ, “Magnetic-field modeling with surface currents. Part I. Physical and computational principles of bfieldtools,” J. Appl. Phys, vol. 128, no. 6, Aug. 2020, Art. no. 063906.

[26] Roth BJ, Sepulveda NG, and Wikswo JP, “Using a magnetometer to image a two-dimensional current distribution,” J. Appl. Phys, vol. 65, no. 1, pp. 361–372, Jan. 1989.

[27] Roth BJ and Wikswo JP, “Apodized pickup coils for improved spatial resolution of SQUID magnetometers,” Rev. Sci. Instrum, vol. 61, no. 9, pp. 2439–2448, Sep. 1990.

[28] Yeo W-J, Yeo Y-R, and Taulu S, “Towards analytical calculation of the magnetic flux measured by magnetometers,” Phys. Lett. A, vol. 411, Sep. 2021, Art. no. 127545.

[29] Sarvas J, “Basic mathematical and electromagnetic concepts of the biomagnetic inverse problem,” Phys. Med. Biol, vol. 32, no. 1, pp. 11–22, Jan. 1987. 3823129 10.1088/0031-9155/32/1/004

[30] Malmivuo J, Suihko V, and Eskola H, “Sensitivity distributions of EEG and MEG measurements,” IEEE Trans. Biomed. Eng, vol. 44, no. 3, pp. 196–208, Mar. 1997. 9216133 10.1109/10.554766

[31] Srinivasan R, Winter W, and Nunez PL, “Source analysis of EEG oscillations using high-resolution EEG and MEG,” Prog. Brain Res, vol. 159, pp. 29–42, Oct. 2006. 17071222 10.1016/S0079-6123(06)59003-X PMC1995013

[32] Lebedev VI and Laikov DN, “A quadrature formula for the sphere of the 131st algebraic order of accuracy,” Doklady Math, vol. 59, no. 3, pp. 477–481, 1999.

[33] Zetter R, Mäkinen AJ, Iivanainen J, Zevenhoven KCJ, Ilmoniemi RJ, and Parkkonen L, “Magnetic field modeling with surface currents. Part II. Implementation and usage of bfieldtools,” J. Appl. Phys, vol. 128, no. 6, Aug. 2020, Art. no. 063905.

[34] Gramfort A , “MNE software for processing MEG and EEG data,” NeuroImage, vol. 86, pp. 446–460, Feb. 2014. 24161808 10.1016/j.neuroimage.2013.10.027 PMC3930851

[35] Shah V, Osborne J, Orton J, and Alem O, “Fully integrated, standalone zero field optically pumped magnetometer for biomagnetism,” Proc. SPIE, vol. 10548, pp. 89–95, Feb. 2018.

[36] Zetter R, Iivanainen J, and Parkkonen L, “Optical co-registration of MRI and on-scalp MEG,” Sci. Rep, vol. 9, no. 1, p. 5490, Apr. 2019. 30940844 10.1038/s41598-019-41763-4 PMC6445124

[37] Iivanainen J , “Calibration and localization of optically pumped magnetometers using electromagnetic coils,” Sensors, vol. 22, no. 8, p. 3059, Apr. 2022. 35459044 10.3390/s22083059 PMC9024658

[38] Bezsudnova Y, Koponen LM, Barontini G, Jensen O, and Kowalczyk AU, “Optimising the sensing volume of OPM sensors for MEG source reconstruction,” NeuroImage, vol. 264, Dec. 2022, Art. no. 119747. 36403733 10.1016/j.neuroimage.2022.119747 PMC7615061

[39] Nenonen J, Kajola M, Simola J, and Ahonen A, “Total information of multichannel MEG sensor arrays,” in Proc. 14th Int. Conf. Biomagnetism (Biomag), 2004, pp. 630–631.

[40] Dale AM and Sereno MI, “Improved localizadon of cortical activity by combining EEG and MEG with MRI cortical surface reconstruction: A linear approach,” J. Cognit. Neurosci, vol. 5, no. 2, pp. 162–176, Apr. 1993. 23972151 10.1162/jocn.1993.5.2.162

[41] Hämäläinen MS and Ilmoniemi RJ, “Interpreting magnetic fields of the brain: Minimum norm estimates,” Med. Biol. Eng. Comput, vol. 32, no. 1, pp. 35–42, Jan. 1994. 8182960 10.1007/BF02512476

[42] Lin F-H, Witzel T, Ahlfors SP, Stufflebeam SM, Belliveau JW, and Hämäläinen MS, “Assessing and improving the spatial accuracy in MEG source localization by depth-weighted minimum-norm estimates,” NeuroImage, vol. 31, no. 1, pp. 160–171, May 2006. 16520063 10.1016/j.neuroimage.2005.11.054

[43] Nurminen J, Taulu S, and Okada Y, “Effects of sensor calibration, balancing and parametrization on the signal space separation method,” Phys. Med. Biol, vol. 53, no. 7, pp. 1975–1987, Apr. 2008. 18354243 10.1088/0031-9155/53/7/012

[44] McPherson A, Fahmy I, Larson E, Yeo W, Holmes N, and Taulu S, “Refined signal space separation methods for on-scalp MEG systems,” Phys. Med. Biol, vol. 70, no. 13, Jul. 2025, Art. no. 135005. 10.1088/1361-6560/ade6ba 40541227

[45] Tierney TM, Seedat Z, St Pier K, Mellor S, and Barnes GR, “Adaptive multipole models of optically pumped magnetometer data,” Hum. Brain Mapping, vol. 45, no. 4, 2024, Art. no. e26596. 10.1002/hbm.26596 PMC10910270 38433646

[46] Boto E , “Triaxial detection of the neuromagnetic field using optically-pumped magnetometry: Feasibility and application in children,” NeuroImage, vol. 252, May 2022, Art. no. 119027. 35217205 10.1016/j.neuroimage.2022.119027 PMC9135302

